# Effect of DNA methylation, modified by 5-azaC, on ecophysiological responses of a clonal plant to changing climate

**DOI:** 10.1038/s41598-022-22125-z

**Published:** 2022-10-14

**Authors:** Veronika Kosová, Vít Latzel, Věroslava Hadincová, Zuzana Münzbergová

**Affiliations:** 1 Department of Botany, Faculty of Science, Charles University, Prague, Czech Republic; 2 Institute of Botany, Academy of Sciences of the Czech Republic, Průhonice, Czech Republic

**Keywords:** Climate-change ecology, Grassland ecology

## Abstract

Epigenetic regulation of gene expression is expected to be an important mechanism behind phenotypic plasticity. Whether epigenetic regulation affects species ecophysiological adaptations to changing climate remains largely unexplored. We compared ecophysiological traits between individuals treated with 5-azaC, assumed to lead to DNA demethylation, with control individuals of a clonal grass originating from and grown under different climates, simulating different directions and magnitudes of climate change. We linked the ecophysiological data to proxies of fitness. Main effects of plant origin and cultivating conditions predicted variation in plant traits, but 5-azaC did not. Effects of 5-azaC interacted with conditions of cultivation and plant origin. The direction of the 5-azaC effects suggests that DNA methylation does not reflect species long-term adaptations to climate of origin and species likely epigenetically adjusted to the conditions experienced during experiment set-up. Ecophysiology translated to proxies of fitness, but the intensity and direction of the relationships were context dependent and affected by 5-azaC. The study suggests that effects of DNA methylation depend on conditions of plant origin and current climate. Direction of 5-azaC effects suggests limited role of epigenetic modifications in long-term adaptation of plants. It rather facilitates fast adaptations to temporal fluctuations of the environment.

## Introduction

The ongoing global climate change represents one of the greatest recent challenges for plants^1,2^. Identification of the mechanisms allowing plants to mitigate negative effects of the change on their performance and survival thus became one of the key recent research topics. Migration, phenotypic plasticity and genetic differentiation have been among the most frequently considered and scrutinized mechanisms^3^. However, there are other mechanisms such as heritable epigenetic variation that can play a potentially important role in plant responses to novel climates that have been studied only scarcely (but see e.g.^3–7^).

Epigenetic regulation of gene expression e.g. via DNA methylation, belongs among important mechanisms allowing species to phenotypically respond to current conditions as well as to conditions experienced by the species in previous years and/or even generations^8^. In contrast to the genetic adaptation, the epigenetic adaptation can arise within a single generation without any underlying genetic change and the epigenetic responses can thus be much faster than the genetic responses^9^. The epigenetic variation can arise for example by silencing of transposable elements and may thus depend on the genetic set-up of the species^10,11^, but can also result from random epimutation^12,13^ or be induced by environmental change^9^. Because environmentally induced DNA methylation variation can be inherited by offspring generations^6^, the epigenetic changes may contribute to species ability to adapt to the rapidly changing climates^14,15^.

One of the common approaches for testing whether the epigenetic inheritance plays a role in the plant adaptation to the climate change is analyzing epigenetic profiles of natural plant populations originating from various environmental conditions exposed to different target conditions. Several studies already provided evidence that the epigenetic variation is correlated with the environmental conditions of plant populations^4,16–19^. Many of these studies, however, do not enable separation of the epigenetic and genetic effects. Studies on natural plant populations separating the epigenetic from genetic differentiation are still quite scarce but highly desirable^7,20–25^.

It has been suggested that particularly adaptive changes in ecophysiological traits related for example to photosynthetic activity and water management should be crucial for maintaining plant fitness under the changing climate^3,26,27^. Ecophysiological traits are often highly plastic and variable among populations (e.g.,^28–35^). Importantly, several ecophysiological traits such as stomata density and/or length have also been shown to be under epigenetic control^36–39^ and can be transgenerationally inherited^40,41^. To what extent is the adaptive ecophysiological differentiation between populations originating from different conditions caused by heritable epigenetic variation, however, remains to be tested. A recently developed method^42^ allowing to modify epigenetic set-up of established plants and thus directly test the importance of epigenetic memory for species response to the changing climate provides unique opportunity to obtain novel insights into the issue.

To better understand the role of epigenetic processes in ecophysiological response of plants to the changing climatic conditions, we used a clonal grass, *Festuca rubra*, as a model. In our previous studies using the same species, we have already demonstrated that the ecophysiological traits such as maximum photosystem II efficiency (F_v_/F_m_), osmotic potential, stomata density and length are highly plastic and/or differ between populations originating from different climatic conditions with stomatal density showing lower levels of plasticity than the other traits^43^. To what extent are the observed patterns epigenetically coordinated remains to be unraveled. The four above mentioned traits have been selected because they are all known to be closely linked to species performance under the changing environmental conditions (e.g.,^30,44–46^). F_v_/F_m_ provides information on overall photosynthetic capacity of the plant^47,48^ and is an indicator of damage in PSII reaction center due to stress^49,50^. Osmotic potential is key drought resistance trait with high level of plasticity^51^. Stomatal density and stomatal length regulate gaseous exchange^30^ and thus are crucial for regulation of photosynthesis^46^ by driving the trade-off between water loss and carbon acquisition by the plant^52,53^.

The relatively simple yet powerful method for testing the role of DNA methylation in response to the environmental change is comparison of genetically identical but epigenetically distinct individuals subjected to different environments^54^. Experimental alteration of DNA methylation status via application of a demethylating agent such as 5-azacytidine (5-azaC) is probably the easiest approach available for plants. 5-azaC inhibits methylation of newly synthetized DNA^55^. A clonal plant continuously exposed to this agent thus produces ramets with altered (reduced) level of DNA methylation^54^.

Demethylation by 5-azaC has been already applied in our model system leading to changes in plant response to the environment^7^. Application of the 5-azaC on the plants in our model system led to changes in some growth- and fitness-related traits and the effects interacted with the conditions of plant origin as well as of plant cultivation suggesting that these traits are controlled epigenetically^7^. Interestingly, plant responses to the conditions of plant origin in terms of growth- and fitness-related traits were stronger in plants treated with 5-azaC than in untreated plants. This could be because the epigenetic memory on the environmental conditions of plant origin was overwritten by the epigenetic adaptations to conditions in the experimental garden, thus equalizing plant performance.

In this study, we used the same plants as Münzbergová et al.^7^ to explore variation in the ecophysiological traits. The plants originate from western Norway and were collected in natural grassland ‘climate grid’ with factorially crossed gradients of temperature and precipitation spanning ~ 4 °C and ~ 2100°mm of precipitation (the SeedClim grid, for details see e.g.,^56^). We cultivated control plants and plants treated with 5-azaC in two growth chambers set to simulate two climatic extremes (cold-dry and warm-wet environment) mimicking the original localities. By cultivating the plants originating from the different original climates under the different cultivation climates in the growth chambers, we simulated different directions and magnitudes of climate change. In addition to exploring variation in the ecophysiological traits, we also explored to what extent the ecophysiological traits may explain variation in the previously measured fitness-related traits.

Specifically, we asked the following questions: (1) What is the effect of experimental application of 5-azaC on the ecophysiological traits?, (2) Do the effects of application of 5-azaC depend on plant origin and cultivating conditions?, (3) Do the effects of application of 5-azaC differ between genotypes and can these differences be explained by their genetic relatedness?, (4) Is there any relationship between the ecophysiological traits and fitness-related traits and does this relationship depend on application of 5-azaC and climate of cultivation?

We hypothesized that: (1) the ecophysiological traits will be significantly influenced by the application of 5-azaC. The effect will be the weakest in the least plastic trait, i.e. stomata density. (2) Plant origin as well as cultivation conditions will strongly interact with the application of 5-azaC. The plants treated with 5-azaC will show higher between population differentiation than the untreated plants due to removal of the epigenetic memory on the common garden conditions experienced during the plant precultivation. (3) The effects of application of 5-azaC will differ between the genotypes and these differences could be explained by their genetic relatedness. The strength of this effect will differ between the different cultivating conditions. (4) The ecophysiological traits will be related to the proxies of plant fitness, but the relationship will depend on the conditions of plant cultivation and the application of 5-azaC.

## Methods

### Study material

*Festuca rubra* L. is a common perennial grass species of temperate grasslands in Europe. *Festuca rubra* used in the experiment is a widespread hexaploid type from the *F. rubra* complex. The species has 42 chromosomes^57^. The species grows across a wide climatic gradient in grasslands, both as a dominant with only few other species and also as a minor component of species rich stands. It reproduces by seeds as well as vegetatively^58^.

The model plants are not under any legal protection, so no permission was required for their study. The plants have been identified by ZM and VH. The material has been studied already in several previous studies and has been characterized also genetically and in terms of genome size. The material has not been deposited in any herbaria. Collection of plant material was in line with relevant institutional, national, and international guidelines and legislation. Specifically, no permissions were required for any of the actions taken.

### Study sites

Plant material originated from a natural climate grid in western Norway (the SeedClim Grid, previously used by e.g.,^56,59–62^). We used plants from the four most extreme localities from this grid. These four localities represent 2 levels of mean annual precipitation (ca. 600 and 2700 mm, later referred to as dry and wet, but note that our dry locality might be considered wet in other regions) and 2 levels of summer temperature (means of the four warmest months, 6.5 °C and 10.5 °C, later referred to as cold and warm). The exact positions of the localities are as follows, warm-dry: 61.035°N, 9.079°E, warm-wet: 60.690°N, 5.965°E, cold-dry: 61.024°N, 8.124°E and cold-wet: 60.934°N, 6.414°E. The communities are grazed intermediate-rich meadows with comparable slopes, grazing regime and bedrock. For more details about the localities, see^56,58^.

The plant material was collected as a part of a previous experiment, data from which were used in several papers^35,43,58,63^, in July 2014. For the current study, we used 6 clones (genotypes) of *F. rubra* collected at each locality at least 1 m apart from each other. We transported the plants and cultivated them in experimental garden of the Institute of Botany, Czech Academy of Sciences, Průhonice, Czech Republic (49.994 N, 14.566 E; means of the four warmest months 16.5 °C; and regular watering during the vegetation season). Microsatellite analysis of the material confirmed that each experimental plant represents an independent genotype^57^.

### Experimental demethylation by 5-azaC application

**Figure 1:**
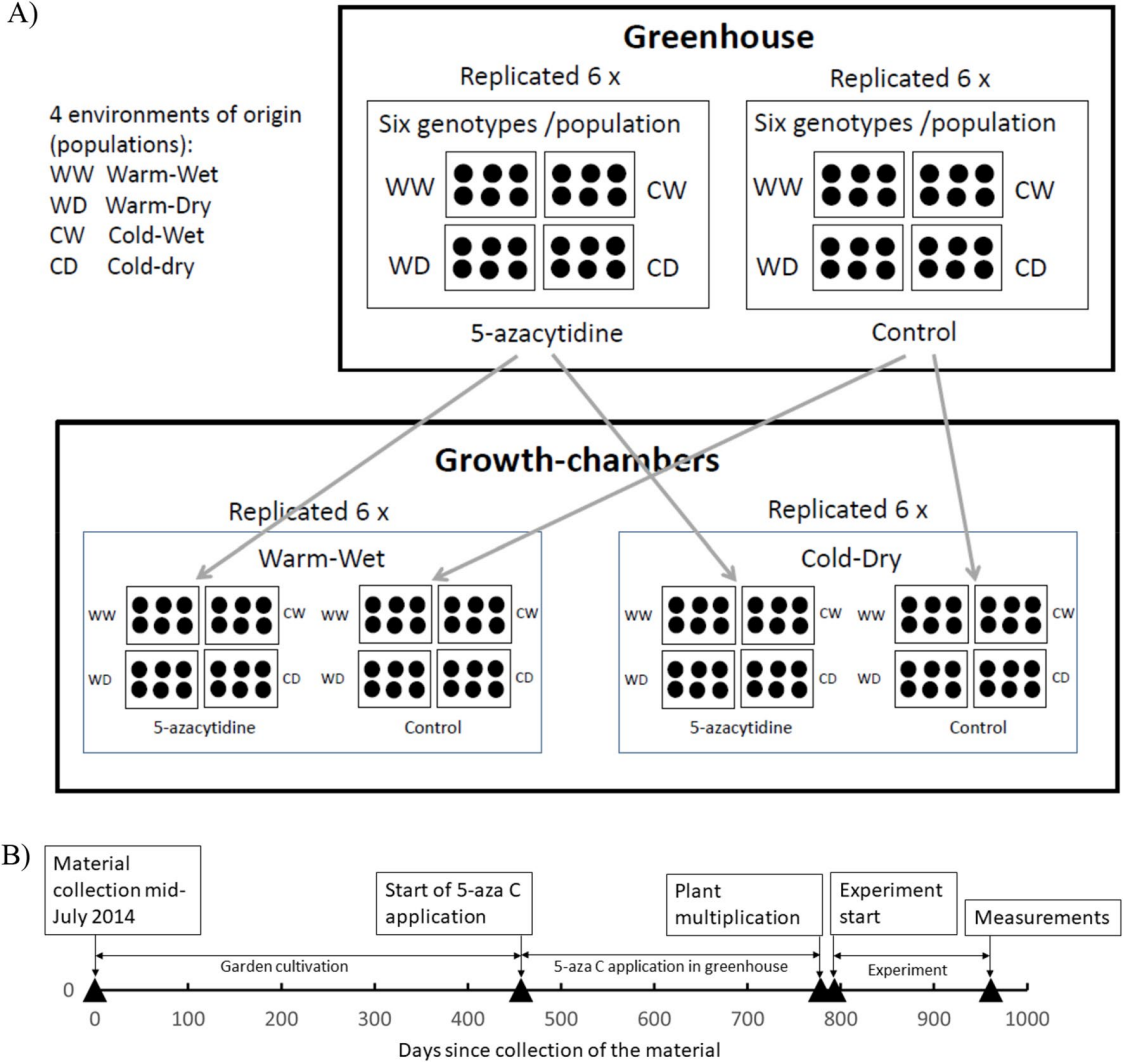
(**A**) The design of the experiment as previously shown in Münzbergová et al.^7^, which provided the material for the current study. (**B**) Timeline of the whole study.

The current study is based on an experiment initially set up for the study of Münzbergová et al.^7^ exploring the effects of 5-azaC treatment on growth- and fitness-related traits (this experiment is independent of the experiment mentioned above). Twelve single ramets (later referred as plants) from each of the 6 genotypes per population were separately planted into 10 × 10 × 10 cm pots in a greenhouse in October 2015. Random half of the plants (six per genotype) was chosen for the 5-azaC application treatment. Each genotype thus had two treatments (5-azaC treated plants and control) with 6 replicates per treatment, i.e. 288 pots in total (6 clones × 6 replicates × 4 populations × 2 5-azaC treatments, Fig. 1). The positions of the plants in the greenhouse have been randomized and randomly changed every month.

The plants were sprayed three times in a week with 100 μmol solution of 5-azacytidine (5-azaC) with added surfactant (Silwet L-77, Lehle Seeds) in one-liter solution for 10 months between October 2015 and August 2016. It has been suggested that the effects of 5-azaC spraying may be long-term due to long-term inhibition of 5-DNA-methyltransferase^64^ and such a long 5-azaC may thus not be necessary. We, however, decided to apply 5-azaC over 10 months as our model species is a relatively slow growing clonal plant, and we wanted to ensure that for the main experiment we have sufficient number of ramets which were produced by mother ramets, themselves arising after the start of the 5-azaC treatment. Control plants were sprayed with water containing surfactant to account for possible confounding effects of the surfactant. Each plant was sprayed with five pulses of a mechanical sprayer to ensure that all plants received comparable amount of 5-azaC^7^. We sprayed the plants in the early morning to ensure that the plants had open stomata and the solution of 5-azaC could be easily absorbed by the leaves^54^. The temperature in the greenhouse was maintained not to drop below 10 °C. The plants were regularly watered.

While it would be useful to measure the exact level of demethylation achieved in our plants by the application of 5-azaC, as done e.g. by Puy et al.^42^, no such information has unfortunately been obtained in our study. In addition, application of 5-azaC may theoretically have other effects than only demethylation. Due to all this, we speak about effects of application of 5-azaC rather than effects of demethylation in the text. Previous studies, however, suggest that the main effect of application of 5-azaC is indeed demethylation of the plants^42^. We thus expect that the 5-azaC application will modify ecophysiology of our model plant and finding such a pattern will indicate that the ecophysiological traits are under epigenetic control (see “Discussion” for more on the issue). The plants did not show any growth abnormalities caused by the 5-azaC treatment.

### Growth chamber experiment

In September 2016, several ramets (each with three leaves and without visible signs of initiating flower stems) were cut from each of the 288 (6 clones × 6 replicates × 4 populations × 2 5-azaC treatments) plants and were placed into small plastic cups filled with water to set roots. After 2 weeks, two ramets from each initial plant were individually planted with developed roots into 5 × 5 × 8.5 cm pots filled with a mixture of common garden soil and sand in 1:2 ratio. The prepared plants were placed into two growth chambers simulating the two strongest climatic extremes along the climatic grid—warm-wet and cold-dry (these conditions were the most extreme as detected in our previous study^58^). The exact setting of the growth chambers is described in Supplementary Table 1. In each growth chamber, there were 6 genotypes, from each of four original localities, receiving the two 5-azaC treatments each originating from 6 separate pots, i.e. 288 plants in each of the two growth chambers, i.e. 576 plants in total. The plants grew in the chambers for 24 weeks. All the plants within a drought treatment received the same amount of water, so we did not account for possible higher moisture requirements of larger plants (but the size differences were relatively low). The positions of the plants in the growth chambers within each moisture treatment have been randomized and randomly changed every month.

It may be argued that our experiment is pseudoreplicated as it was performed only in 2 growth chambers and the growth chambers may theoretically differ in a range of other variables (e.g. light intensity) leading to possible spurious treatment effects^65^. As we already pointed out in our previous studies^58,66^, the conclusions of Hurlbert^65^ on pseudoreplication in growth chamber experiments have been extensively criticized (e.g.^67,68^). In fact, also Hurlbert^69^ concluded that such experiments can be analyzed with standard statistical approaches as long as the interaction term is used as an estimate of the error term to test the main effect. In our experiment, we were not primarily interested in the effect manipulated at the growth chamber level, i.e. the cultivating conditions. We were primarily interested in the effect of plant origin and 5-azaC application, which are well replicated and also in the interaction between the plant origin, 5-azaC application and cultivating conditions. In such a case, using the standard error terms is well justified. Thus, in line with a range of other studies using similar settings such as unreplicated gardens at different elevations^70,71^ or growth chambers^72–76^, we suggest that such studies are useful to study various factors possibly interacting with the climate of cultivation as done here.

### Species traits

After 24 weeks, when the plants reached reasonable size to be measured, we measured the ecophysiological photosynthesis-related traits: maximum photosystem II efficiency (parameter F_v_/F_m_), osmotic potential, stomatal density and stomatal length. Maximum photosystem II (PSII) efficiency (F_v_/F_m_, commonly referred to as chlorophyll fluorescence) was measured using FluorPen FP-100 MAX/USB (Photon System Instruments, Czechia) in dark-acclimated (1 h) plants (Maxwell and Johnson 2000). In healthy leaves, F_v_/F_m_ value is usually close to 0.8. Lower values indicate that a proportion of PSII reaction centers is damaged or inactivated leading to photoinhibition^78^.

Osmotic potential at full turgor was determined psychrometrically from leaf sap using dew point microvoltmeter Wescor HR-33T (Wescor, Inc., USA) with C-52 sample chambers as described previously^43,79^. All plants have been fully saturated by water for 24 h prior to the sampling. Afterwards, two young but fully developed leaves of each plant were put into a syringe, sealed, and stored frozen at − 20 °C. At the time of measurement, the samples were defrosted at room temperature, released cytoplasmic solution was squeezed into the syringe tip and 7 µL of the solution was pipetted onto a filter paper disc placed in 1.25 mm deep sample holder of the C‐52 chamber. Voltage reading was taken after 6 min, which was found to be sufficient time for vapor pressure equilibration. Voltage was converted to osmotic potential using 0.3 M NaCl standard of − 1.37 MPa.

Stomatal density and stomatal length were measured using epidermal impressions of the adaxial side of the leaf blade using a clear nail polish. The impressions were mounted on microscope slides with a transparent adhesive tape. Stomatal density was counted as an average of three no overlapping areas (each 500 × 500 µm). Each counting area was in contact with a margin of the leaf to avoid possible variation in the stomata size from the leaf margin to the center. From each of these three areas, three stomata were randomly chosen, and their length was measured, resulting in 9 measured stomata of each sample. Preliminary explorations of the stomatal imprints indicated low variation in the stomata across the leaf margin suggesting that the 9 stomata measured at the leaf margin well represent the stomata of the given leaf (Hadincová pers. obs.). We did not measure the stomata in the leaf centers as the leaves are naturally folded and the stomata are hard to observe in the folded parts with the occurrence of ribs.

To explore the relationship between the physiological traits and plant fitness, we used number of ramets and aboveground biomass, measured in our previous study^7^ as proxies of fitness. While it is generally assumed that lifetime fitness should be represented e.g. by lifetime seed production^80^, our model is a long-lived species with extensive clonal reproduction and measuring lifetime fitness is not straightforward. The two traits used here as proxies of fitness are expected to be closely related to seed production and vegetative spread of the individuals^58,81^. These data are visualized in Supplementary Fig. 1.

### Data analysis

To estimate the effect of application of the 5-azaC, population of plant origin, genotype, growth chamber and all their interactions on all the dependent variables, we used linear model using function lm with type III sum of squares in R version 4.0.3^82^. Genotype was nested within population of origin in all the tests (formula: lm (trait ~ (Population/ Genotype) × 5-azaC × Growth chamber)). The data on stomata density and size and maximum photosystem II efficiency (F_v_/F_m_) fitted the assumption of the model for normality and homoscedasticity of the data. The data on osmotic potential did not fit the assumption and could not be easily transformed to achieve normality. We thus multiplied the values by -1 (as the values are negative). After this transformation, these data fitted Gamma distribution and could be analysed with the formula glm (trait ~ (Population/ Genotype) × 5-azaC × Growth chamber, family = “Gamma”).

Then, we explored dependence of differences in the response to the application of 5-azaC between the genotypes on genetic relatedness of the genotypes^58,83^. For this purpose, we calculated pairwise genetic relatedness (calculated as Rho/(1-Rho) between studied genotypes) using data on five microsatellite markers identifying 63 different alleles in the samples^57^. To calculate differences in the response of the different genotypes to the application of 5-azaC, we calculated average proportional change in each trait between the 5-azaC treated genotypes and the controls. For that we used average trait values for each genotype in each chamber across the 6 replicates. From these values, we generated matrix using Euclidean distance among the plants for each growth chamber and for each trait separately. Then we tested correlation between the matrix of genetic distances and the matrix of distances in response to application of 5-azaC using Mantel test as implemented in R package vegan^84^, formula: mantel (matrix 1, matrix 2, permutations = 999). The tests were done for each growth chamber and each trait separately. To check whether the differences observed could be due to the genetic differentiation in the performance of the control plants, we repeated the test using difference in traits between the control plants. This later test was never significant (p > 0.1 in all the traits).

Because the relationships between the genetic similarity and the similarity in response to the application of 5-azaC could be driven by between population differences, we also performed additional tests using partial Mantel tests. Here, we used a matrix of populations of origin (coded 0 for pair of genotypes which do not come from the same population and 1 for pair of genotypes which come from the same population) as a covariate (formula: mantel.partial (matrix 1, matrix 2, matrix covariate, permutations = 999)). If this relationship was significant, we repeated the test using trait values of control plants only (as described above). This control test was never significant (p > 0.1 in all the traits).

Finally, we tested phenotypic selection of the ecophysiological traits. For these analyses, both ecophysiological traits and proxies of fitness (number of ramets and aboveground biomass) were standardized to a mean of zero and a unit variance across the whole dataset (after sqrt transformation of the fitness proxies to achieve normality and homogeneity of variance). We then tested the effect of the physiological traits alone and in interaction with growth chamber and 5-azaC application on proxies of plant fitness (formula: lm (fitness proxy ~ (ecophys. trait1 + ecophys. trait2 + ⋯) × 5-azaC × Growth chamber). Due to a range of significant interactions, we also calculated these relationships separately for each growth chamber and 5-azaC application treatment and report standardized directional selection gradients (β), calculated as a partial regression coefficient from multiple linear regressions to relative values of proxies of fitness on all standardized ecophysiological traits^85^, in the results separately for each 5-azaC treatment and growth chamber. Because the selection effects may not be linear but rather quadratic^86^, we added the quadratic version of the traits into our models and tested if the models improved. As the models did not improve in any case (based on AIC), we do not report these results further.

### Ethical approval

The model plants are not under any legal protection, so no permission was required for their study. The plants have been identified by ZM and VH. The material has been studied already in several previous studies and has been characterized also genetically and in terms of genome size (see “Methods”). The material has not been deposited in any herbaria. Collection of plant material was in line with relevant institutional, national, and international guidelines and legislation. Specifically, no permissions were required for any of the actions taken.

## Results

### Effect of 5-azaC application and its interactions with other factors

**Figure 2:**
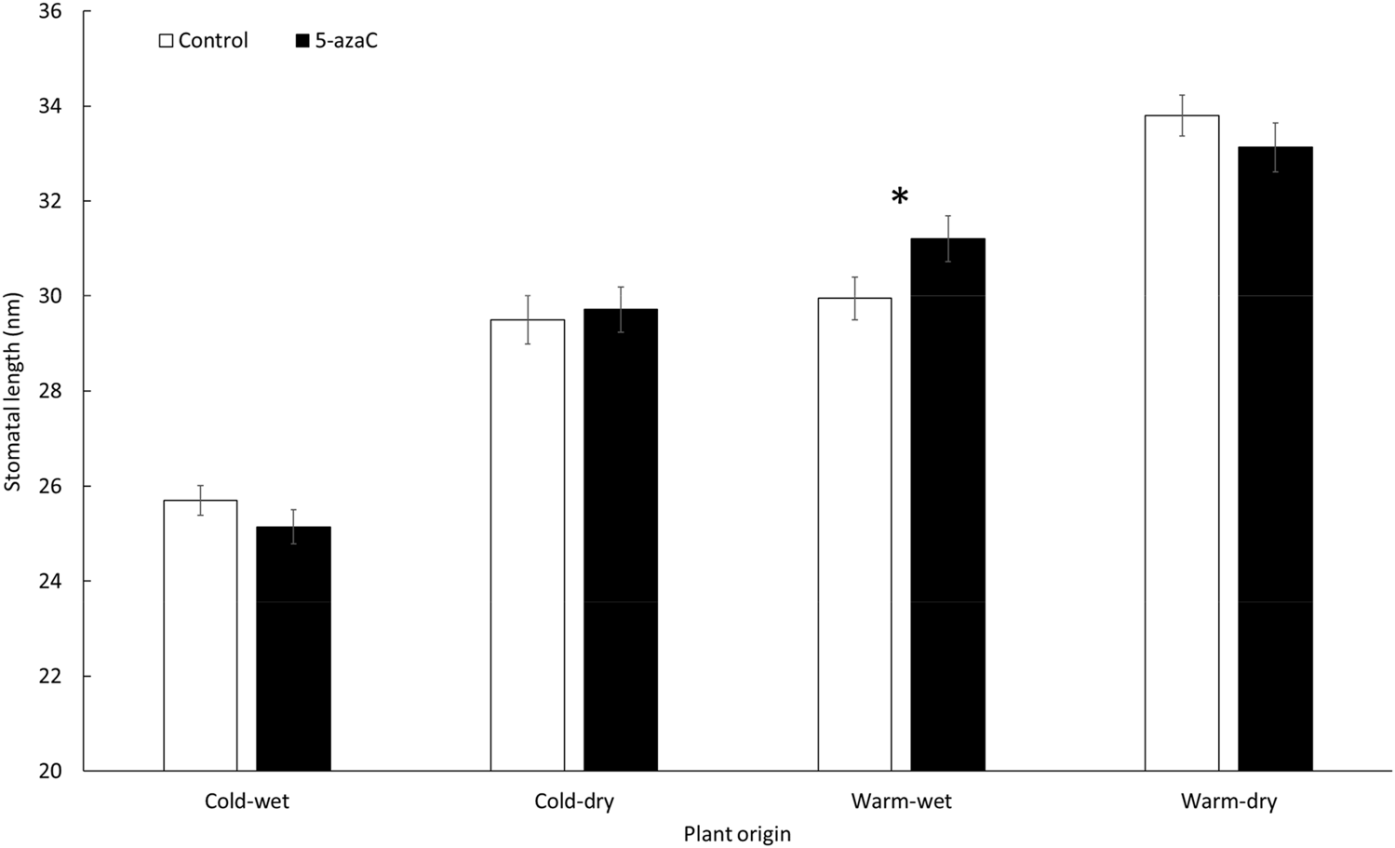
The effect of interaction between original conditions and 5-azaC application on stomatal length. Columns show mean ± SE. Asterix denotes significant difference within pair of columns (p ≤ 0.05). The figure is shown as a box plot in Supplementary Fig. 2. The data were measured at the end of the experiment using 6 replicates of each of 6 genotypes from each of 4 populations of origin grown in 2 growth chambers, all subjected to control and 5-azaC treatments, resulting in 576 experimental plants. The overall test is reported in Table 1.

Plants of different genotypes and localities of origin differed in all the measured traits (Supplementary Figs. 3, 4). Plants also differed in response to cultivation conditions (growth chambers) in all the measured traits but stomata density (Table 1). We did not detect any main effect of 5-azaC on any of the measured trait, but its effects interacted with the other variables in three cases (Table 1). Specifically, the effect of 5-azaC on length of stomata depended on plant origin (Table 1, Fig. 2). Plants from the warm-wet locality and treated with 5-azaC had longer stomata than controls (on average by 1.25 μm, i.e. 4%), but no significant effect of 5-azaC was detected in the plants from the other localities (Fig. 2).

**Table 1:** The effect of 5-azaC application, growth chamber, population of origin and genotype and their interactions on F_v_/F_m_, osmotic potential, stomatal density and stomatal length in the growth chambers.

	df	Fv/Fm		Osmotic potential		Stomatal density		Stomatal length	
		F value	p value	F value	p value	F value	p value	F value	p value
5-azaC	1	1.44	0.231	0.57	0.45	0.52	0.471	0.03	0.862
Growth chamber	1	15.58	** < 0.001**	174.1	** < 0.001**	2.35	0.126	94.16	** < 0.001**
Population	3	55.59	** < 0.001**	31.98	** < 0.001**	28.00	** < 0.001**	162.2	** < 0.001**
Genotype in population	20	6.65	** < 0.001**	2.43	** < 0.001**	5.39	** < 0.001**	8.06	** < 0.001**
5-azaC × chamber	1	1.52	0.218	0.09	0.765	0.04	0.849	0.02	0.883
5-azaC × popul	3	0.27	0.847	0.79	0.501	0.88	0.451	2.83	**0.038**
5-azaC × genotype	20	0.77	0.753	1.02	0.434	1.21	0.237	1.62	**0.044**
Chamber × popul	3	10.93	** < 0.001**	14.54	** < 0.001**	3.39	**0.018**	4.30	**0.005**
Chamber × gen	20	2.83	** < 0.001**	1.42	0.108	1.43	0.102	1.42	0.109
5-azaC × chamber × popul	3	3.75	**0.011**	0.31	0.82	1.61	0.187	0.35	0.791
5-azaC × chamber × gen	20	1.44	0.1	1.33	0.152	0.77	0.747	1.06	0.393
Residuals		474		458		472		477	

Genotype was nested in population of origin. Significant values (p ≤ 0.05) are in bold.

**Figure 3:**
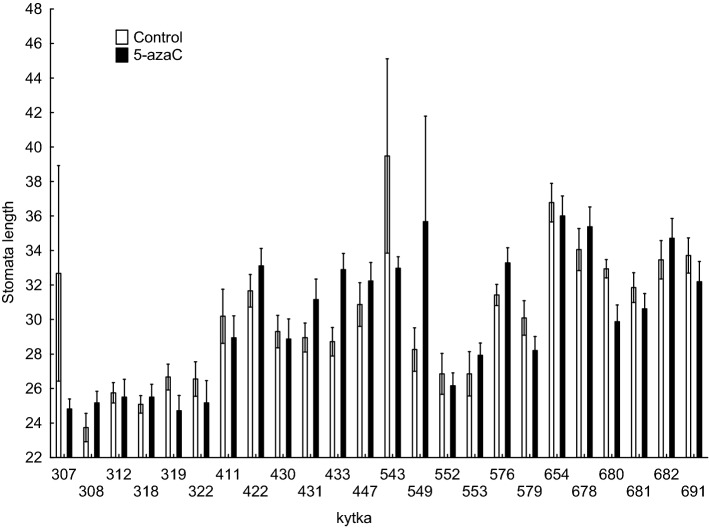
Differences in response to 5-azaC application among different genotypes. Each pair of bars represents one genotype. The genotypes are grouped by their environment of origin. Pairs of columns marked with * are significantly (p < 0.05) different from each other. The data were measured at the end of the experiment using 6 replicates of each of 6 genotypes from each of 4 populations of origin grown in 2 growth chambers, all subjected to control and 5-azaC treatments, resulting in 576 experimental plants. The overall test is reported in Table 1.

The effect of 5-azaC application on stomatal length also differed among the genotypes (Table 1). When tested separately by genotype, the control and 5-azaC treated individuals of four genotypes (out of 24 genotypes in the experiment) differed significantly in stomatal length with the highest difference of 4.18 μm between the treatments. Out of these, two showed significant increase in the stomatal length after 5-azaC (one genotype from cold-dry and one from warm-wet locality) and the other two significant decrease (one from cold-wet and one from warm-dry, Fig. 3).

**Figure 4:**
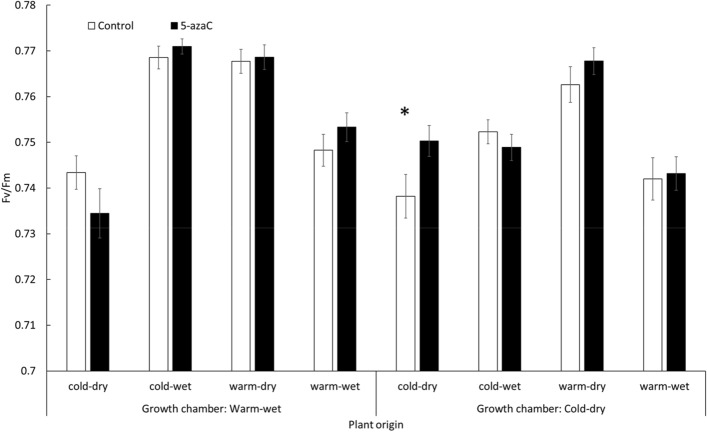
The effect of interaction between original conditions, growth chamber and 5-azaC application on maximum photosystem II (PSII) efficiency (F_v_/F_m_). Columns show mean ± SE. Asterix denotes significant difference within pair of columns (p ≤ 0.05). The data were measured at the end of the experiment using 6 replicates of each of 6 genotypes from each of 4 populations of origin grown in 2 growth chambers, all subjected to control and 5-azaC treatments, resulting in 576 experimental plants. The overall test is reported in Table 1.

For maximum photosystem II efficiency (F_v_/F_m_), a significant triple interaction of 5-azaC, plant origin and growth chamber was detected (Table 1). Plants originating from the cold-dry locality treated with 5-azaC and grown in the cold-dry growth chamber had significantly higher F_v_/F_m_ (by ± 0.012 on average) than the corresponding control plants. No significant difference was detected in any other combination (Fig. 4).

### Effects of genetic relatedness on response to 5-azaC application

We found significant positive correlation between the genetic relatedness of the genotypes (pair-wise Rho/(1-Rho)) and their response to the application of 5-azaC for F_v_/F_m_ in the cold-dry growth chamber. The result became slightly stronger when the population of origin was added as a covariate (Table 2). No significant relationships with the genetic relatedness have been detected for the other traits (Table 2).

**Table 2:** Correlation between genetic relatedness of the genotypes and their similarity in response of each trait to 5-azaC application for each growth chamber separately.

Chamber	cov	Fv/Fm		Osmotic potential		Stomatal density		Stomatal length	
		r	p	r	p	r	p	r	p
Warm–wet	–	0.02	0.423	0.06	0.200	0.04	0.277	0.01	0.443
	Pop	− 0.01	0.534	0.08	0.134	0.04	0.263	− 0.05	0.715
Cold–dry	–	**0.19**	**0.005**	− 0.14	0.966	0.05	0.274	− 0.08	0.885
	Pop	**0.21**	**0.008**	− 0.14	0.962	0.05	0.249	− 0.07	0.862

All the tests were done without a covariate (cov.) as well as with population of origin as a covariate. Significant values (p ≤ 0.05) are shown in bold.

### Relationship between ecophysiological traits and fitness proxies

Both the ramet number and the aboveground biomass were significantly negatively related to F_v_/F_m_ in the overall tests (main effect—M in Table 3). The aboveground biomass also increased with increasing stomatal length and density. The other main effects of the physiological traits on the fitness proxies were not significant (Table 3). In most cases, the effects of physiological traits interacted with the cultivating conditions, with the relationships being generally stronger in the warm-wet than in the cold-dry growth chamber (Table 3). Only the effect of F_v_/F_m_ on aboveground biomass significantly interacted with the 5-azaC application, with the relationship between F_v_/F_m_ and fitness proxies being stronger in the plants receiving 5-azaC than in the control plants (treatment—T in Table 3). In neither of the tests, we detected significant triple interaction between the physiological traits, cultivating conditions and 5-azaC application (Table 3).

**Table 3:** Relationships between ecophysiological and fitness-related traits in control plants and plants treated with 5-azaC growing in two different environments in growth chambers (warm-wet and cold-dry).

Growth chamber	Signif	Warm-wet		Cold-dry	
Treatment		Control	5-azaC	Control	5-azaC
**Number of ramets**					
Fv/Fm	M, GC	**− 0.35**	**− 0.24**	0.07	0.05
Osmotic potential		**0.47**	0.02	**− **0.07	0.13
Stomatal length	GC	**0.22**	0.02	0.09	**− 0.28**
Stomatal density	GC	**− **0.14	**− **0.11	0.09	0.04
**Aboveg. biomass**					
Fv/Fm	M, GC, T	**− 0.31**	**− 0.41**	0.09	**− **0.08
Osmotic potential	M, GC	**− **0.1	**0.58**	**− **0.16	**− **0.02
Stomatal length	M, GC	**0.4**	0.13	0.14	**− 0.22**
Stomatal density		**0.15**	0.07	0.02	0.06

The table shows standardized linear selection gradients (β) estimated as the multiple regression coefficients of relative fitness (i.e. standardized ramet number and aboveground biomass) on standardized mean trait values (ecophysiological traits). Significant β values (p ≤ 0.05) are marked in bold. F_v_/F_m_—maximum photosystem II efficiency. Column signif. indicates significant main effect of the trait (M), interaction between the trait and growth chamber (GC), 5-azaC treatment (T) or their interaction (GC × T) when analyzing all the data within a single test.

## Discussion

Our study suggests that the variation in the ecophysiological traits was driven primarily by genetic differences among the plants in mean trait values (the effects of population and genotype), their plasticity (the effects of growth chamber) as well as by the genetic differentiation in trait plasticity (the interactions of growth chamber with population and genotype). In contrast, there was no significant main effect of the application of 5-azaC and only three interactions of the application of 5-azaC with the other factors suggesting relatively limited effect of 5-azaC, and thus likely of DNA methylation, on these traits. The interactions of the climate of origin and cultivation with 5-azaC indicate that species response to the changing climatic conditions is modified by 5-azaC.

### Effects of 5-azaC application

For stomatal length, we found that the 5-azaC treated plants originating from the warm-wet locality had longer stomata than the controls from the same locality. Interestingly, these controls had comparable stomata length as plants originating from very contrasting environment: cold and dry. It is an intriguing finding because longer stomata are usually considered to be selected for in warm and moist environments due to their increased transpirational cooling ability^87^. If the DNA methylation played a role in species adaptation to the conditions of plant origin, we should observe longer stomata in the control plants than in the plants treated with 5-azaC and not the opposite. Similarly as for the photosynthetic efficiency (discussed below), the 5-azaC application thus changed the plant response in non-intuitive direction.

The general expectation if DNA methylation plays a positive role in local adaptation and 5-azaC reduced the DNA methylation level would be better performance of the plants that experience their home environment and their DNA is naturally methylated over genetically identical individuals that were treated by the 5-azaC^88^. In terms of the ecophysiological traits, this would mean that the control plants should show values corresponding to the optimal values in the given conditions, while the 5-azaC treated plants would deviate from this optimal value. This assumption is based on the idea that some of the epigenetic markers related to the long-term adaptation to the conditions of plant origin would be removed and/or changed due to the effect of 5-azaC, which would be observable as a maladaptive response in their home environment. Nonetheless, we observed the opposite response, the value of stomatal length for the given plant origin moved in the direction towards the optimal value in 5-azaC treated plants in comparison to the controls. The response going in an opposite direction than expected is in line with our previous study on growth- and fitness-related traits of the same plants^7^.

One of the possible explanations for the observed patterns could be linked with the design of our study. We cultivated selected plants in a common garden and subsequently in a greenhouse for 2 years before we performed the experiment. This was done in order to let the plants grow to obtain sufficient number of ramets for the experiment. Hence, it is plausible that the plants already lost their epigenetic memory on the field conditions and became epigenetically adjusted to the new experimental conditions^7^. This is in line with other studies indicating that epigenetic memory can be gradually lost over several asexual generations^89,90^. Epigenetic adjustments of the plants to our common garden and greenhouse could be however maladaptive when plants were moved to the growth chambers simulating their original environments. Therefore, the 5-azaC application could disrupt some of the epigenetic memory that reflected the precultivation period and these plants could perform better than the plants with the memory of precultivation (controls). This assumption is supported by Gonzalez et al.^54^ who demonstrated that clonal offspring of *Trifolium repens* grew the same phenotypes as their drought stressed parents unless they were treated via 5-azaC that removed the memory on the drought. After 5-azaC treatment, the plants grew the same phenotype as the offspring of the not stressed parents. An alternative explanation may be that the 5-azaC application changing DNA demethylation can lead to both activation or deactivation of genes underlying the ecophysiological traits^38,91–93^. Therefore, it is possible that demethylation enhances the expression of some genes instead of suppressing it^94^.

The observed effect of the 5-azaC on stomata length is in line with Tricker et al.^39^. They demonstrated that stomatal development under different environmental conditions can be induced by changes in DNA methylation status due to the climate change. It was also found that the stomatal traits could be transgenerationally heritable under environmental stress in *Arabidopsis thaliana* in response to drought^41^ and their development was shown to be under epigenetic control^95^. Stomata traits could also be genetically adapted to different climatic conditions of plant origin^45,96,97^. In Kosová et al.^43^ as well as in this study, we also found genetic differentiation of our populations in the stomatal traits. Our results thus suggest that the expression of the stomatal traits is probably driven by both, genetic as well as epigenetic mechanisms, dependent on the specific climatic conditions of their cultivation.

For photosynthetic efficiency, the effect of 5-azaC was observed in higher photosynthetic efficiency of the plants treated with 5-azaC originating from the cold-dry environments grown in the same conditions when compared to the control plants. Such response does not reflect species long-term epigenetic adaptations to the climate of origin because the plants with natural methylation patterns (controls) should be better adapted to their original climate than the genetically identical plants treated with 5-azaC if epigenetic variation plays a role in local adaptation. It can be explained in the same way as discussed above for the stomatal length.

The effect of 5-azaC on photosynthetic efficiency is in line with Jueterbock et al.^98^ indicating that the photosynthetic traits are closely linked to the variation in DNA methylation in a clonal sea grass. In contrast, others demonstrated that the photosynthetic efficiency was affected by transgenerational memory of drought stress, but this effect was not linked to the changes in the methylome^99,100^. The existing knowledge is thus largely ambiguous and more research is needed in this area, especially for extreme climatic conditions, where damage of photosystem II and whole functioning of photosynthesis apparatus is crucial for plant survival^101,102^.

Despite the high genetic differentiation and plasticity in the osmotic potential shown in this as well as in the previous studies^51,103–105^ we did not find any effect of the 5-azaC on osmotic potential. In contrast to this, Hao et al.^106^ indicated that species have an ability of epigenetic adjustments to osmotic stress. Similarly, Colaneri and Jones^107^ indicated that methylome is highly responsive to the changes in osmotic potential. None of these studies, however, provided direct functional link between the osmotic potential of a plant and the DNA methylation patterns.

Epigenetic variation depends on the specific genetic profiles of the different plants^12,14,108^ but the epigenomes can also be more closely aligned to the local environmental variation than to the genotypic variation^18,83,109^. In our study, response to the 5-azaC application was genotype specific, but genetically more related plants showed more similar response to the 5-azaC only for F_v_/F_m_. According to these results (genotypic as well as the environmental origin response to 5-azaC), we suggest that the response to 5-azaC, and thus DNA methylation patterns, were determined both by the specific genetic structure as well as the local environments^7,110–112^.

### Relationship of ecophysiological and fitness proxies

We expected that the observed changes in the ecophysiological traits translate into changes in plant fitness indicating potential selection on these traits^43,113,114^. This expectation was supported by the significant main effects of the traits in half of our tests. In line with Carlson et al.^31^, we found that the direction and intensity of the relationship depend on the specific conditions with cultivating conditions having much stronger effect than the 5-azaC application. The highest number of relationships was detected in the warm-wet conditions and the control plants and the lowest in cold-dry conditions. While it could be generally expected that the warm-wet conditions are less stressful for plants than the cold-dry conditions, it may be the opposite in our system of alpine species growing along a moisture gradient from very wet to wet, rather than wet to dry. A higher number of significant correlations in the warm-wet conditions may thus be in line with an expectation that the variation in ecophysiological traits plays a role for our fitness proxies (plant biomass and number of ramets) only if the plants are under some level of stress^43,115–117^. These significant correlations may allow evolutionary adaptations of the plants to changing climatic conditions^63^.

The F_v_/F_m_ value was the only parameter showing significant negative relationship with both number of ramets and biomass in the overall test. However, an opposite relationship would be expected—the higher values of F_v_/F_m_, indicating lower damage of the photosystem, the higher plant fitness^117–121^. However, Madriaza et al.^122^ found such a positive relationship only in plants damaged by herbivores, while no such relationship has been found in control plants. The negative relationship shown here seems to be in contrast to all previous findings and may indicate a trade-off in the plants. They either invest into growth at the expense of lower quality of the photosynthetic apparatus or vice versa.

In stomatal traits, the only significant main effect was positive effect of stomatal length on aboveground biomass. In other cases, the effects of stomatal traits interacted with cultivating conditions. Plant fitness proxies were higher in case of longer stomata in control warm-wet chamber, while fitness proxies decreased with stomatal length in cold-dry chamber in plants receiving 5-azaC treatment. Few larger stomata are expected to be less costly than many smaller stomata and can indicate that water supply is optimal in these conditions^123^. The opposite relationship in cold-dry chamber in 5-azaC treated plants may be caused by differential variation in the trait values between the control and 5-azaC treated plants. Alternatively, this may mean that the relationship is affected by some other traits affected by 5-azaC application (note that we did not detect effect of 5-azaC application on stomatal density and for stomata length we only detected significant interaction of 5-azaC with population and genotype) due to trait interactions^124^. This result may also indicate that control plants with naturally methylated DNA perform better when stomata are bigger and fewer possibly suggesting that DNA methylation may have an adaptive value. We, however, also found even a stronger negative relationship between stomatal length and number of ramets in 5-azaC treated plants from the cold-dry chamber. While this supports the general assumption that stomata size and density are important determinants of fitness^125,126^, its specific effects are clearly dependent on actual conditions^43^ and likely affected by DNA methylation.

Increasing values of fitness proxies with increased osmotic potential indicates close link between the traits^43,127^ at least under some conditions. Low osmotic potential indicates accumulation of osmotic solutes in the plants e.g. as a protection against drought or frost^51^. The positive relationship thus indicates that the less stressed plants, not needing to invest to lowered osmotic potential, could invest into growth.

### Methodological considerations

In the study, we assumed that the application of 5-azaC leads primarily to DNA demethylation but did not collect our own data to support the assumption. We suggest that the assumption is reasonable as it is generally accepted that the 5-azaC reduces DNA global methylation through the effect on DNA methyltransferases, an enzyme that bonds methyl group on DNA^128,129^. We have also provided an evidence in our previous studies that the foliar application of 5-azaC reduces overall DNA methylation level by 4–30% in treated *Trifolium repens, Taraxacum brevicorniculatum* and *Fragaria vesca* plants^42,54,130^. In a grass, *Oryza sativa*, foliar application of 75 µM 5-azaC solution reduced their overall DNA methylation level by more than 50%^131^. This indicates that the 5-azaC methylation works even in monocots, and may be even stronger than in dicots, and is thus likely to work in our monocot species as well. In rare circumstances, 5-azaC can induce hypermethylation of particular loci^132^, hence likely silence some genes, but the overall effect of 5-azaC is reduction of DNA methylation level, which is in most cases followed by upregulation of previously epigenetically silenced genes. Therefore, we suggest that the majority of the 5-azaC effects in our study can be ascribed to its demethylating effect than to some other, less likely functions of the 5-azaC on DNA methylation status of plants and on other plant functions.

While we detected significant effects of the 5-azaC application on ecophysiology of the plants (in interaction with other factors), the effects were relatively weak and highly variable. This is likely due to the fact that the 5-azaC is randomly incorporated into the DNA instead of cytosine during its replication resulting in reduced activity of DNA methyltransferase, which is followed by hypomethylation at random sequences and thus random demethylation patterns^133–135^. Multiple rounds of the 5-azaC application over the period of 10 months as done here may have even increased the noise in the system. This could lead to low statistical power of our study as we only worked with 6 genotypes per climate of origin, i.e. 24 genotypes in total (note that this is much higher than most other previous experimental studies on epigenetics as summarized by McGuigan et al.^136^). On the other hand, 5-azaC application has been applied to each genotype in 6 replicates, reducing the chance that the effects detected per genotype are just consequences of the random demethylation. Overall, the fact that we did detected effects of 5-azaC application after accounting for our hierarchical model structure indicates that the 5-azaC application had some predictable effects in our system.

Another important question is whether the effect of 5-azaC applied 6 months before measurements could still be effectively altering ecophysiological traits in our study. There is no general consensus on how persistent the effect of 5-azaC on DNA methylation is over time. 5-azaC induced demethylation of DNA was shown to be passed to progeny^137^ and from seeds to flowering several months later^138^ suggesting long lasting effect of the 5-azaC on the DNA methylation. On the other hand, Kumpatla and Hall^139^ reported only temporary effect of the 5-azaC on the DNA methylation. Although we have no empirical data to support our assumption, we consider it to be very unlikely that the DNA methylation status of the 5-azaC treated plants would completely restore to its original status, notably the DNA methylation patterns induced by the original environment. Hence, considering also the slow growth of our plant species and the detected 5-azaC effects, we assume that the 5-azaC was still effective when we assessed the ecophysiological traits.

The possible suspicion that our experiment is pseudoreplicated as defined by Hurlbert^65^ is extensively discussed in the methods part of the paper.

## Conclusion

Ecophysiological traits of *F. rubra* were not strongly affected by the 5-azaC application. In few cases, we, however, found significant effects of the 5-azaC application on the ecophysiological traits under specific conditions of cultivation of plants of a specific origin. Under the assumption that the 5-azaC reduced the level of DNA methylation, the effects detected indicate that the DNA methylation does not mediate long-term adaptations of the species to their environment of origin. These findings do not support increasing body of evidence that heritable epigenetic differentiation can be an important driver of plant adaptation and/or population differentiation reflecting long-term natural conditions^4,140–143^. In contrast, our results suggest that the heritable epigenetic differentiation, modified by the 5-azaC application, may be an important driver of species response to conditions of a previous season^7,54,66^, allowing for fast responses to the rapidly changing conditions. This is in line with McGuigan et al.^136^ suggesting that epigenetic mechanisms may be enhancing fitness extremely rapidly. This fast ability to epigenetically adjust to the environment may be adaptive in case the conditions repeat in time, but will likely be maladaptive in case of increased variation in climate.

Epigenetic regulation of ecophysiological processes thus seems to play a limited role in the long-term adaptations of plants. However, as the 5-azaC is blocking the function of 5-methyl cytosine and methyl cytosine is an endogenous mutagen in DNA, the sites of DNA methylation are mutational hotspots^144,145^. It is thus possible, that despite our conclusion of the lack of an effect of DNA methylation in the long-term adaptation of plants, the increased level of mutations in the methylated sites may be important for these adaptations.

The observed variation in the ecophysiological traits translated into differences in our fitness proxies (plant biomass and number of ramets) but the effects were context dependent. In addition, most of these effects point to an opposite direction than expected indicating that the ecophysiological traits-fitness proxies’ relationships are very complex. Further studies need to attempt obtaining more mechanistic insights into the epigenetic regulation of the different ecophysiological traits as well as into the trait-fitness proxies’ relationships. These studies always need to be performed under different conditions to account for the strong context-dependency.

## Supplementary Information

Supplementary Information.

## Data Availability

The datasets used and analyzed during the current study are available from the corresponding author on reasonable request.
