# Supplementary material for: Effect of DNA methylation, modified by 5-azaC, on ecophysiological responses of a clonal plant to changing climate
Source: Sci Rep. 2022 Oct 14;12:17262. doi: 10.1038/s41598-022-22125-z (PMC9568541; doi:10.1038/s41598-022-22125-z)

**Supplementary Table 1.** The specific regime settings in the growth chambers providing information on minimum (min), maximum (max) and average (av.) temperature in the growth chamber each day. The regimes mimic the course of temperatures at the localities during the day as well as over the course of the growing season. The dry regime plants were watered with 20 mL of tap water per plant applied to the trays if the soil moisture was lower than 15% (monitored using TMS dataloggers, TOMST co. (Wild et al. 2019). In the wet regime, plants were cultivated under full soil saturation with about 1.5 cm of water in the bottom of the tray. Soil moisture was monitored continuously during the whole experiment and watering was modified to ensure constant moisture throughout the experiment. Five data-loggers were placed in each growth chamber. Each data-logger was placed in a pot with a growing Festuca plant, which was intermixed among the experimental plants and was of the same size as the experimental plants, but was not a part of the experiment. For all the regimes, the same day length and radiation were used, i.e. 16 h of full light (06.00–22.00 h) and 4 h of full dark with a gradual change in light availability in the transition between the light and dark period over 2 h. Over the full light period, the radiation was 360 µmol m^-2^ s^-1^, red radiation (R, k = 660 nm) of 26 µmol m^-2^ s^-1^ and far-red radiation (FR, k = 730 nm) of 15 µmol m^-2^ s^-1^, R/FR = 1.73 (the radiation measured using a SPh 2020 photometer from Optické dílny, Turnov, Czech Republic). This setting corresponds to setting used already in our previous studies (Münzbergová, Hadincová, Skálová and Vandvik 2017).

|  | Cold regime | | | | Warm regime | | |
| --- | --- | --- | --- | --- | --- | --- | --- |
| Time (day) | Min (°C) | Max (°C) | Av (°C) | Min (°C) | | Max (°C) | Av (°C) |
| 1–4 | 5 | 15 | 9.8 | 5 | | 16 | 10.1 |
| 5–25 | 3 | 12.5 | 7.5 | 3 | | 16 | 9.2 |
| 26–46 | 3 | 12.5 | 7.5 | 3 | | 18.5 | 10.2 |
| 47–67 | 3 | 12.5 | 7.5 | 3 | | 24.3 | 12.5 |
| 68–88 | 3 | 14.5 | 8.4 | 3.4 | | 25 | 12.9 |
| 89–176 | 3 | 14.7 | 8.5 | 5 | | 23.8 | 14.8 |

**Supplementary Figure 1.** A) Number of ramets and B) aboveground biomass, used as measures of fitness in this study, and their variation among 5-azaC treatment (C: control, A: 5-azaC), plant origin and growth chambers. The data come from the paper Münzbergová et al. (2019).

**
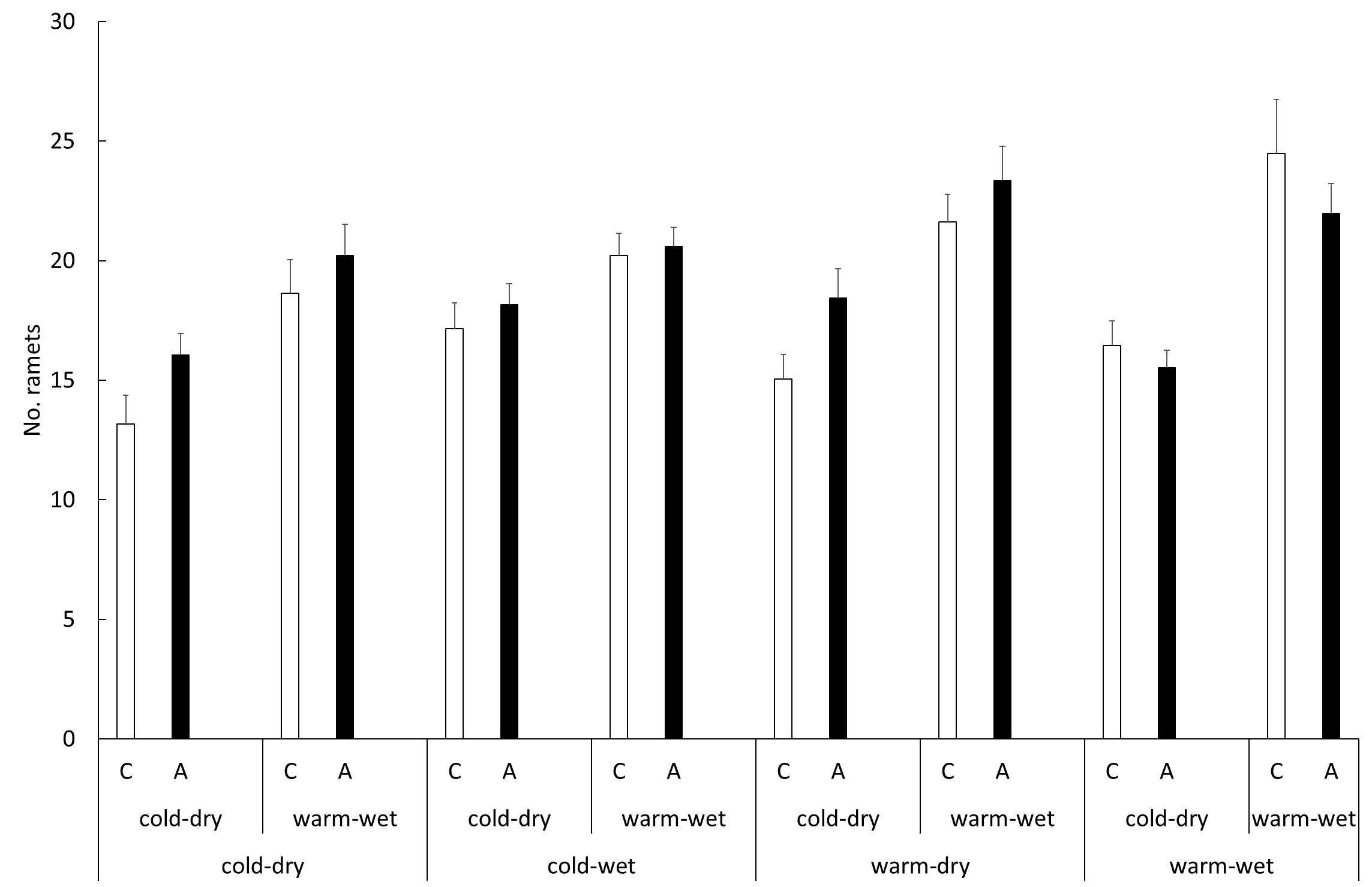
**

**B)**

**
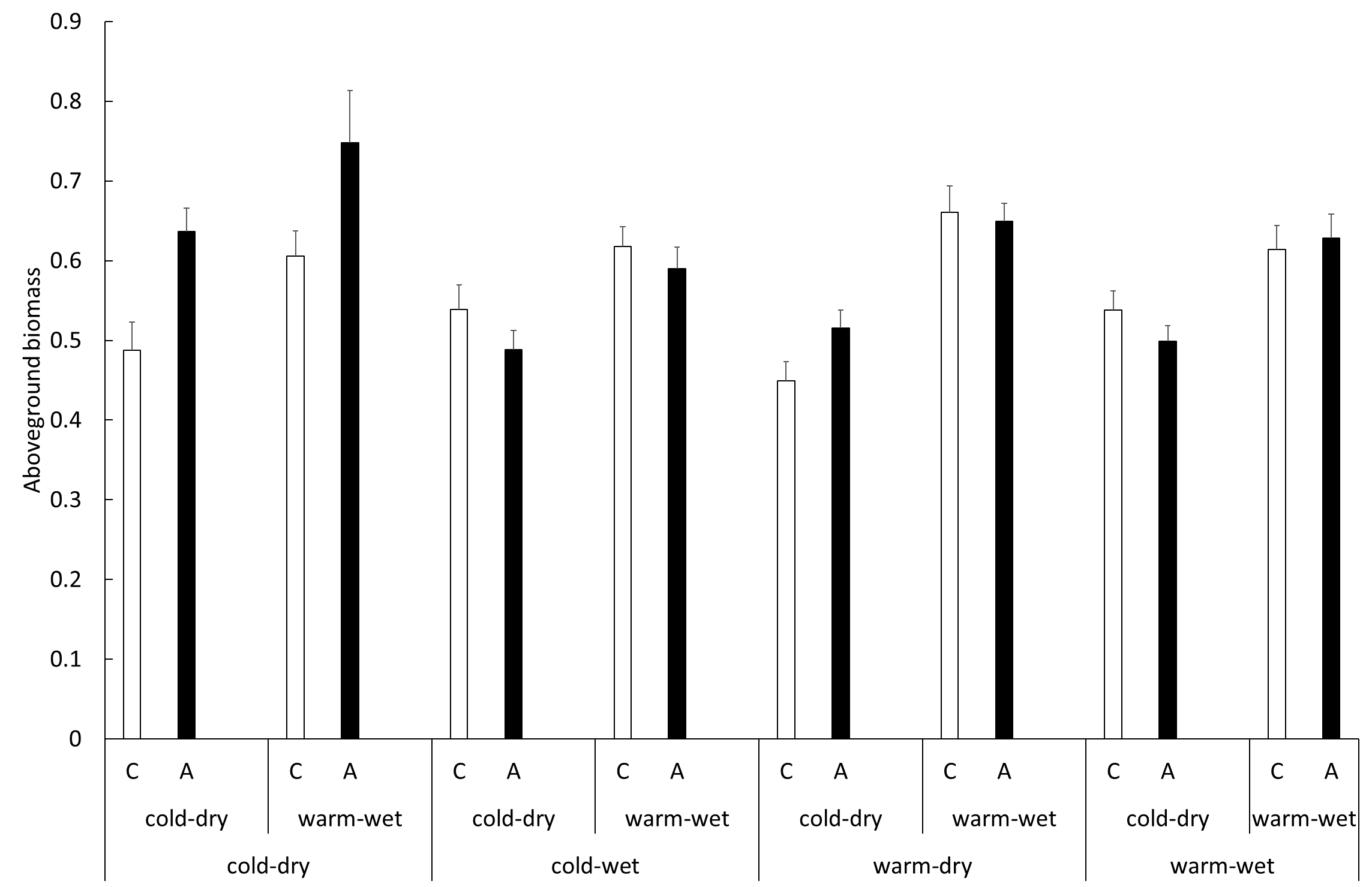
**

**Supplementary Figure 2.** The effect of interaction between original conditions and 5-azaC application on stomatal length. The figure is identical to Figure 2, here shown as boxplot. The data were measured at the end of the experiment using 6 replicates of each of 6 genotypes from each of 4 populations of origin grown in 2 growth chambers, all subjected to control and 5-azaC treatments, resulting in 576 experimental plants.


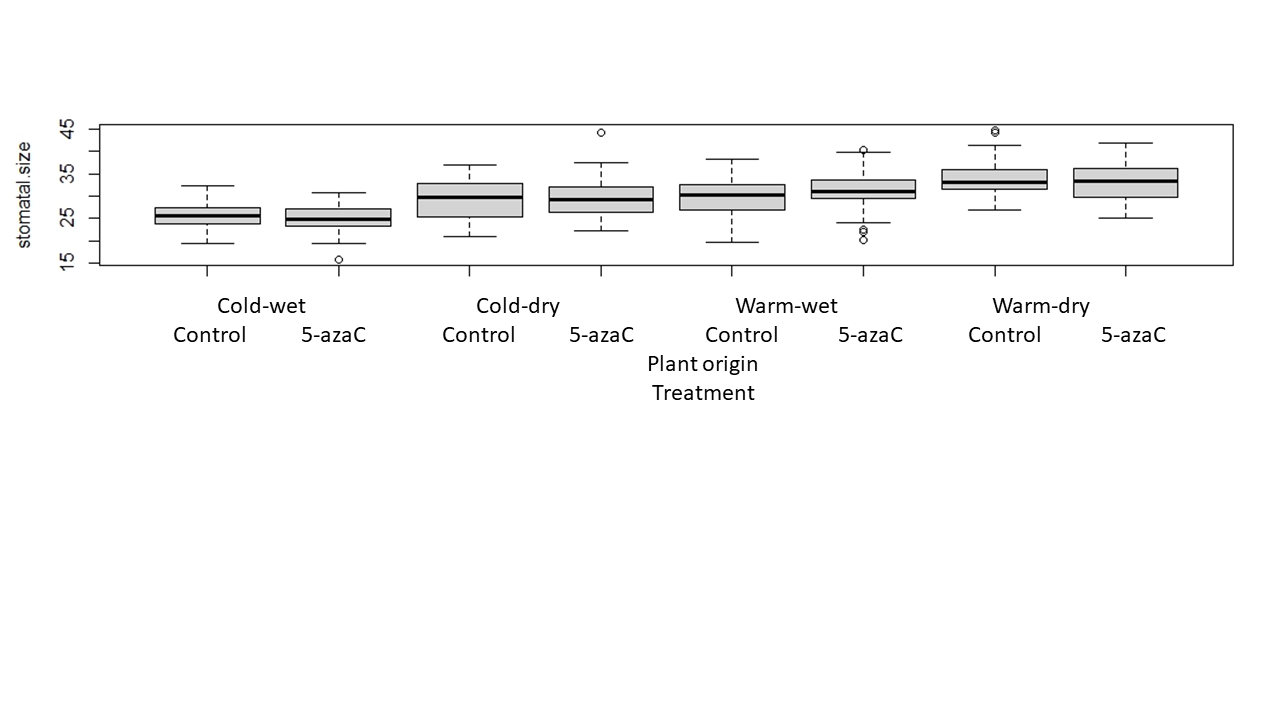


**Supplementary Figure 3.** Mean values of the traits for each genotype and 5-azaC treatment in each growth chamber. A) F_v_/F_m_, B) Osmotic potential, C) Stomatal density, D) Stomatal length, E) Number of ramets and F) Aboveground biomass. The data were measured at the end of the experiment using 6 replicates of each of 6 genotypes from each of 4 populations of origin grown in 2 growth chambers, all subjected to control and 5-azaC treatments, resulting in 576 experimental plants. The data on number of ramets and aboveground biomass come from Münzbergová et al. (2019). Each thin line represents one genotype, the think line represents the average.

A)


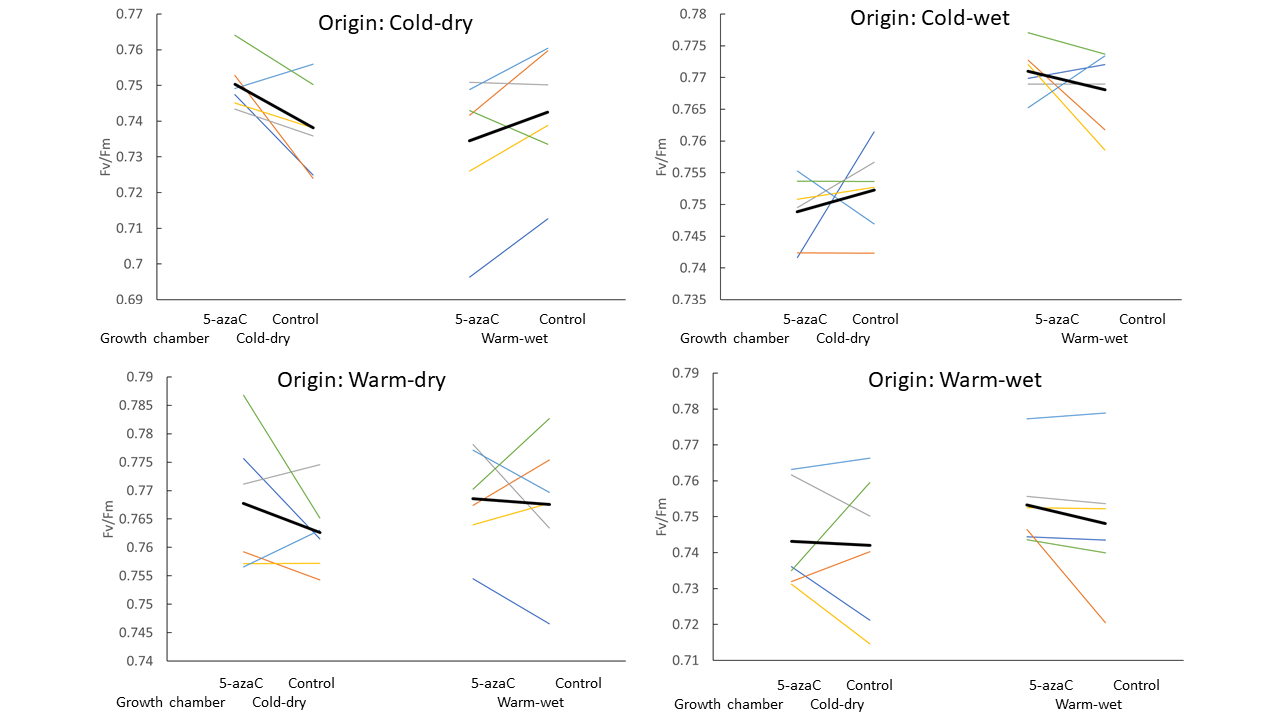


B)


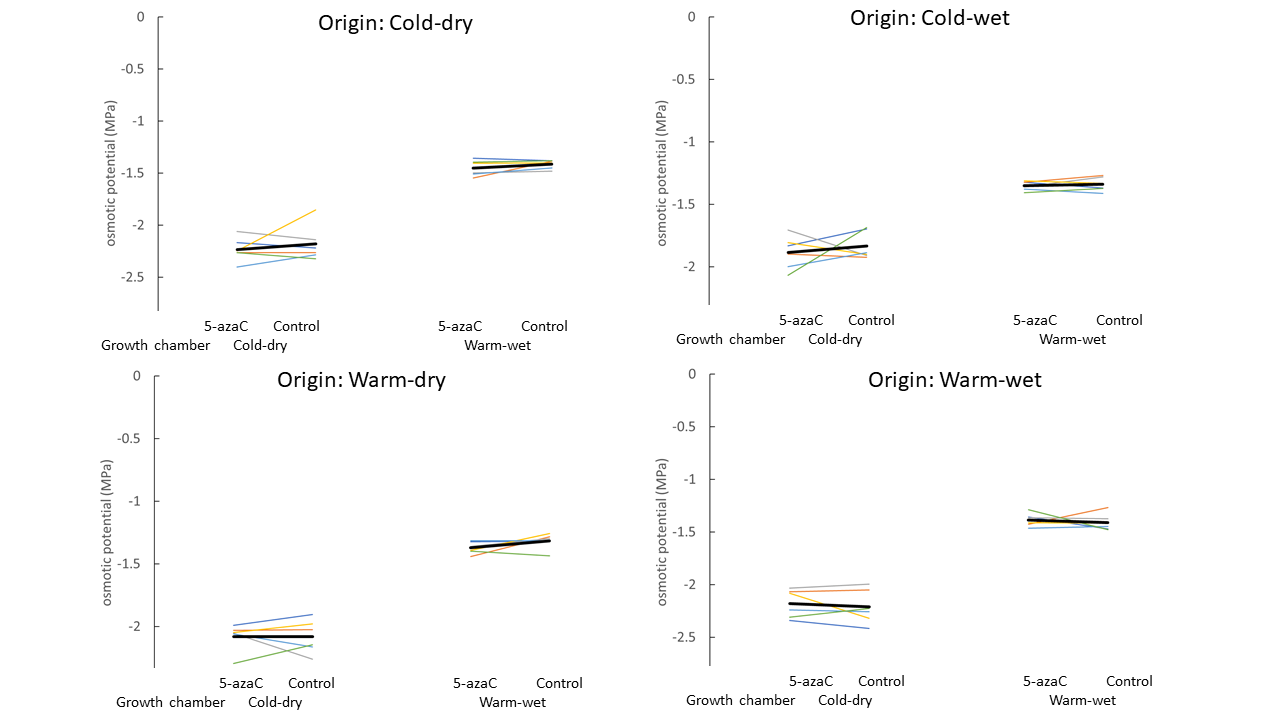


C)


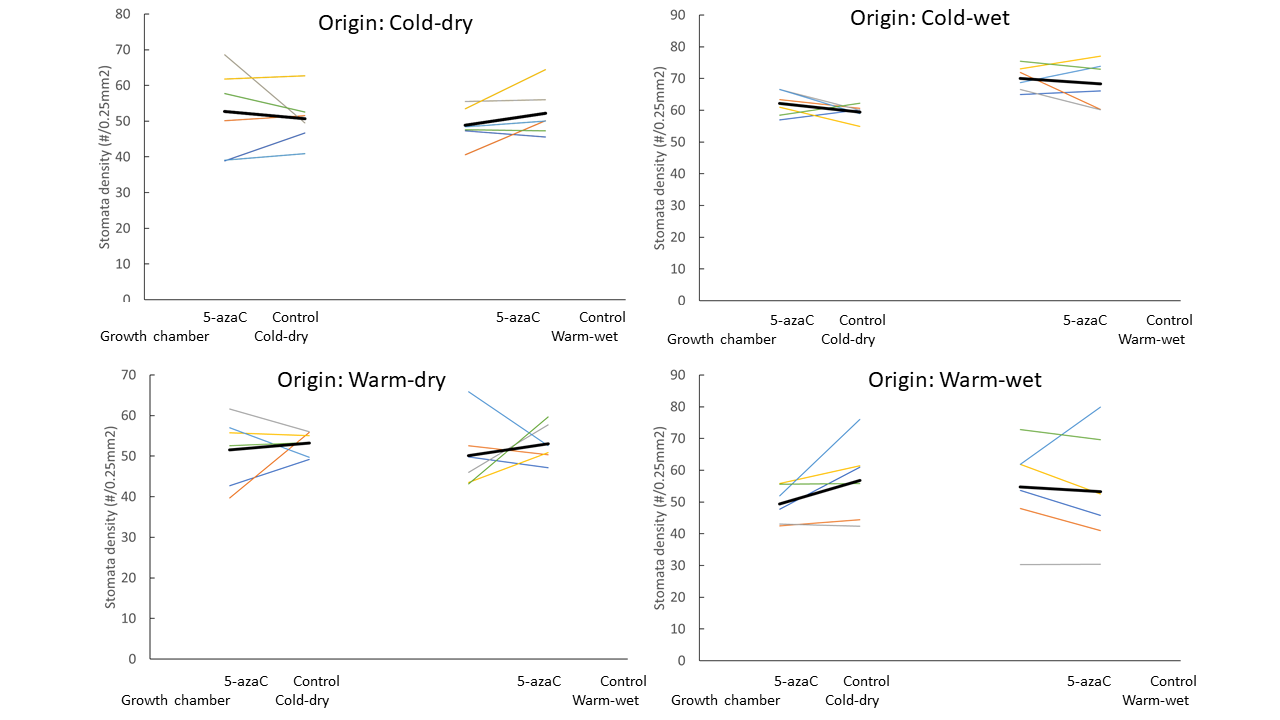


D)


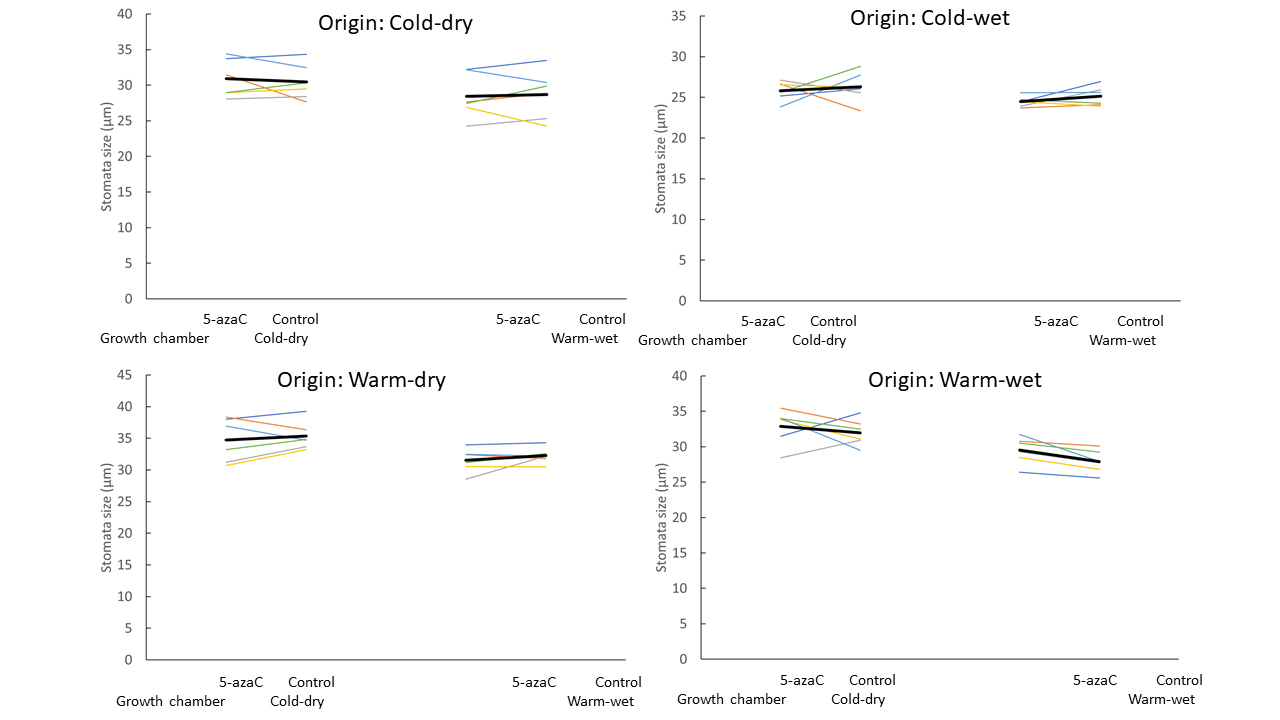


E)


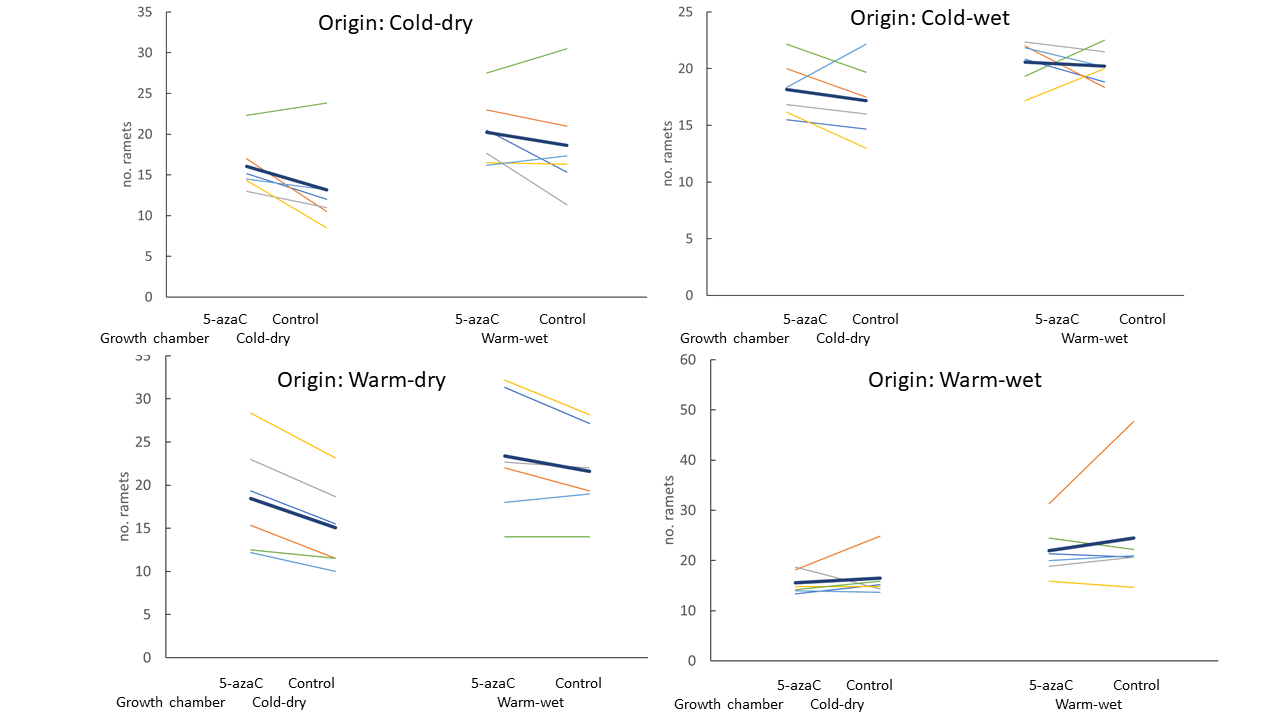


F)


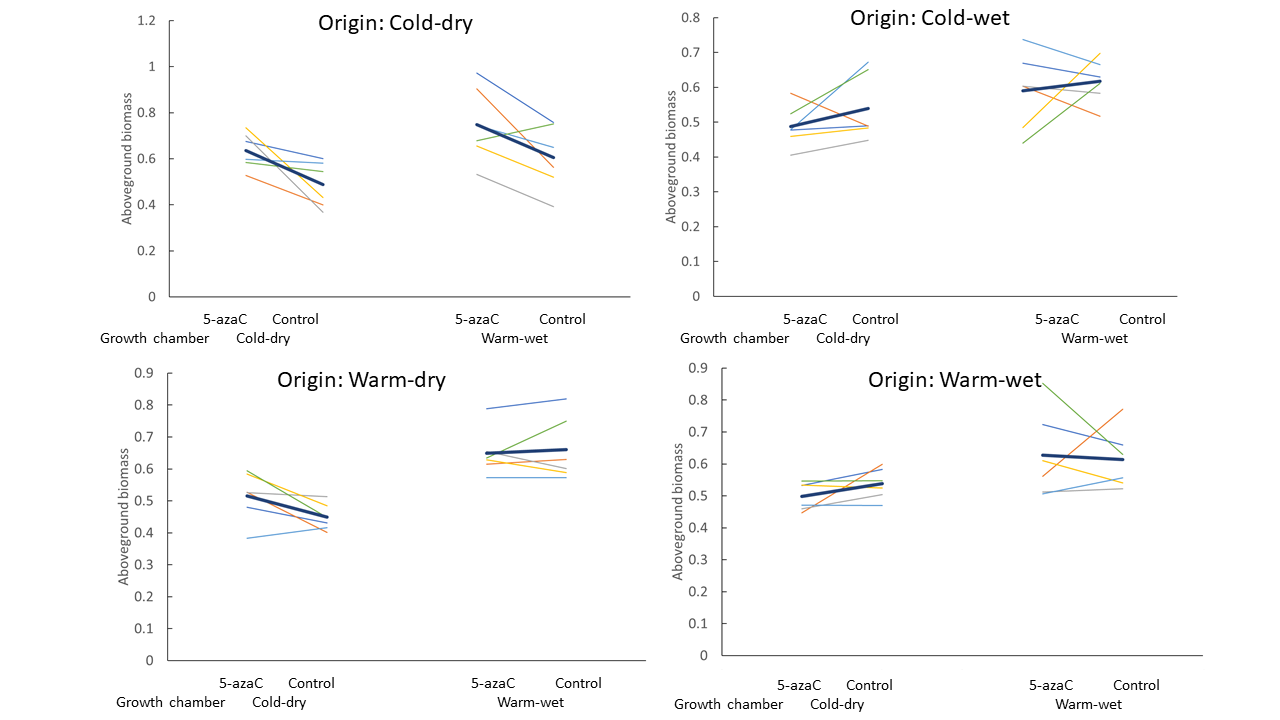


**Supplementary Figure 4.** Mean and SE values of each trait value for each genotype from each origin in each growth chamber and 5-azaC treatment. A) F_v_/F_m_, B) Osmotic potential, C) Stomatal density, D) Stomatal length, E) Number of ramets and F) Aboveground biomass. The data were measured at the end of the experiment using 6 replicates of each of 6 genotypes from each of 4 populations of origin grown in 2 growth chambers, all subjected to control and 5-azaC treatments, resulting in 576 experimental plants.


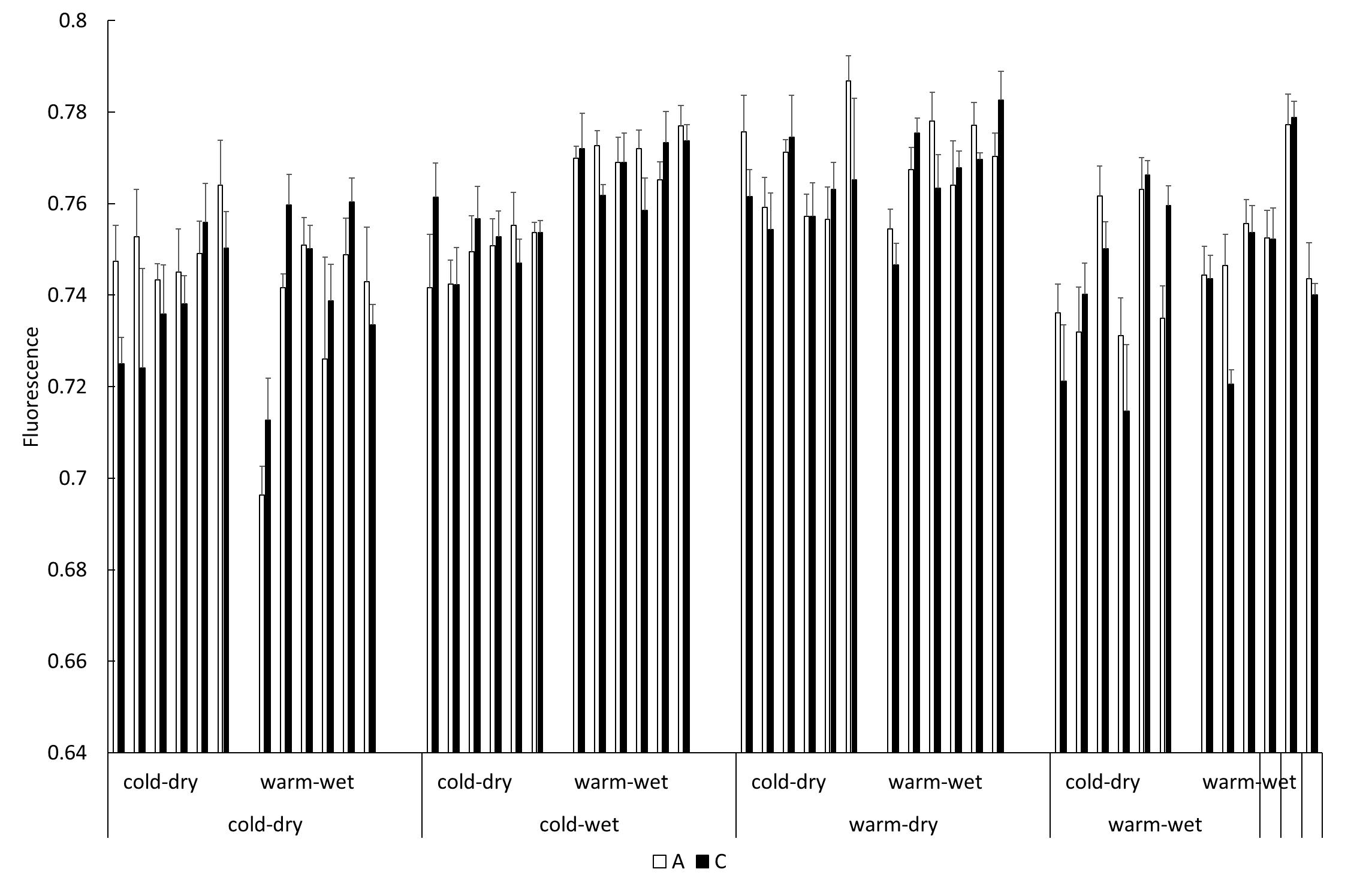


B)


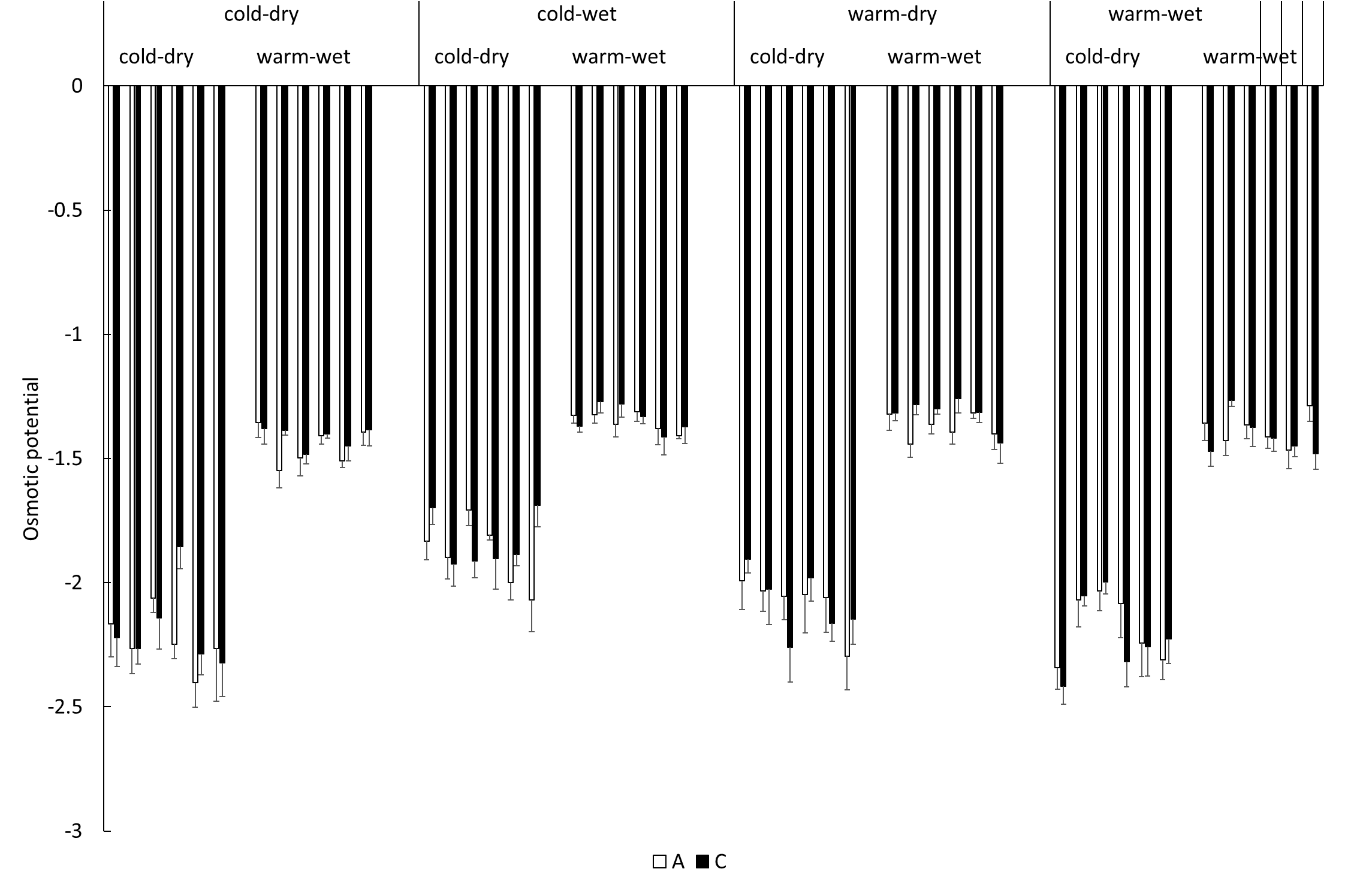


C)


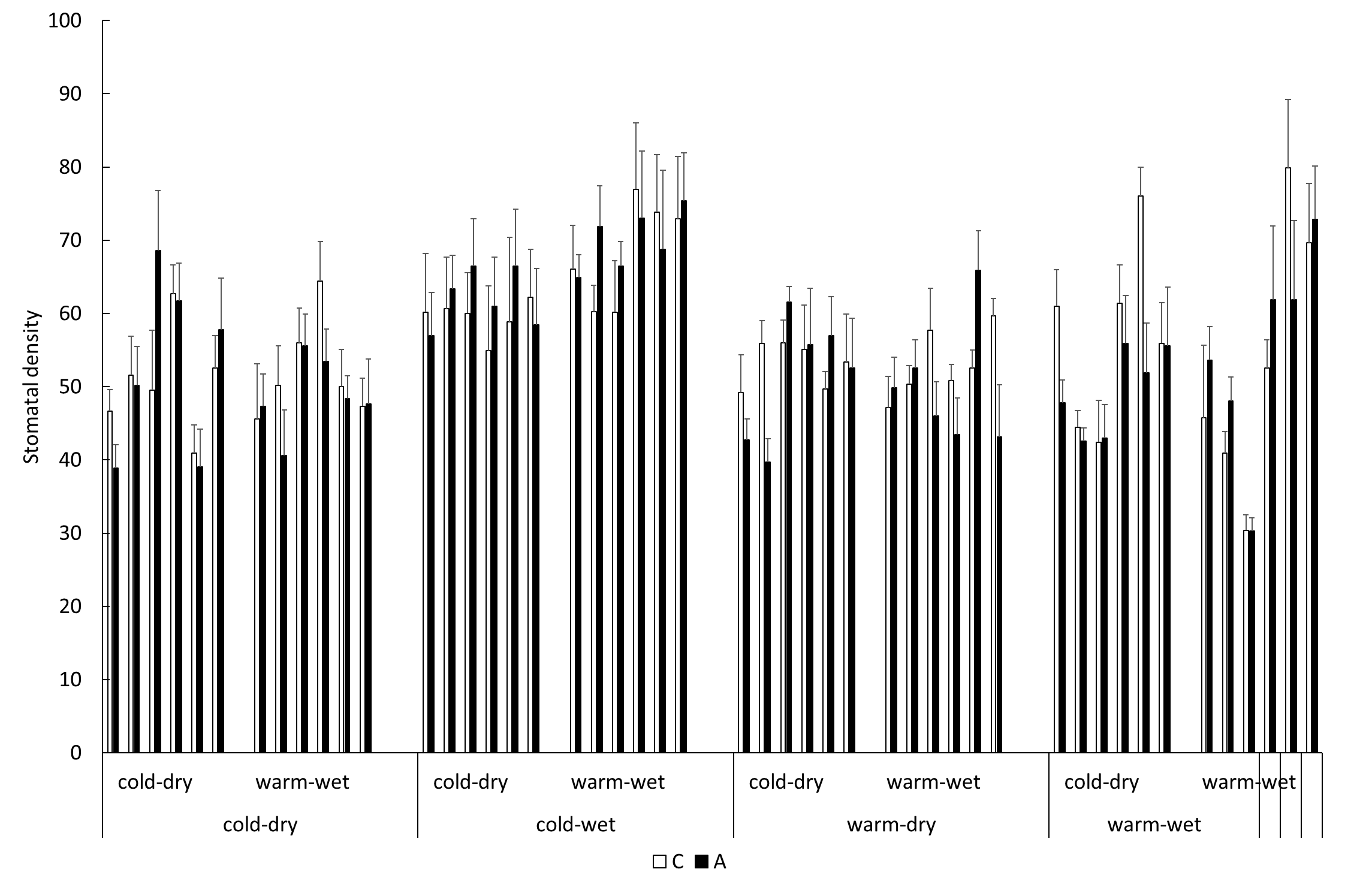


D)


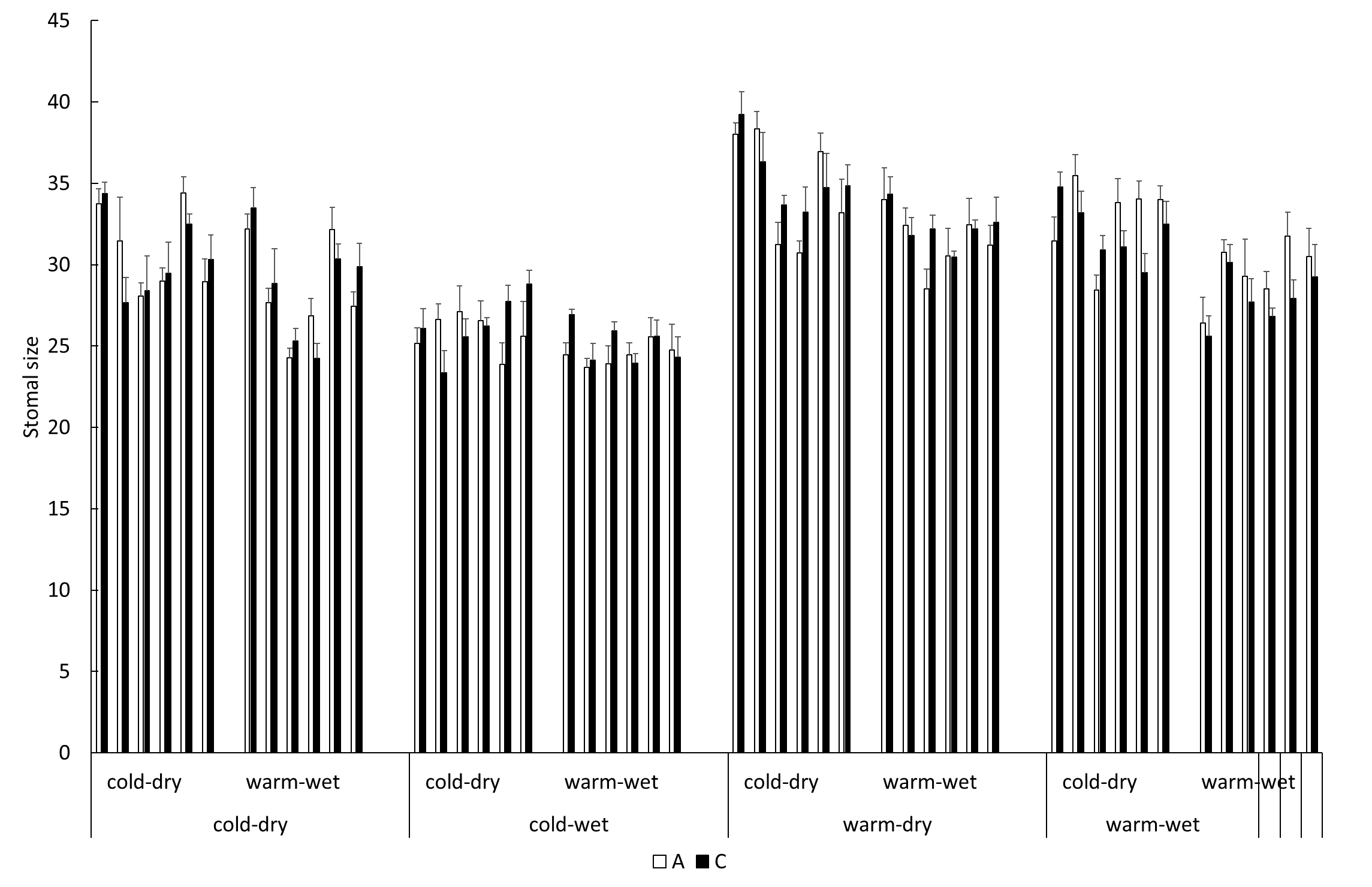


E)


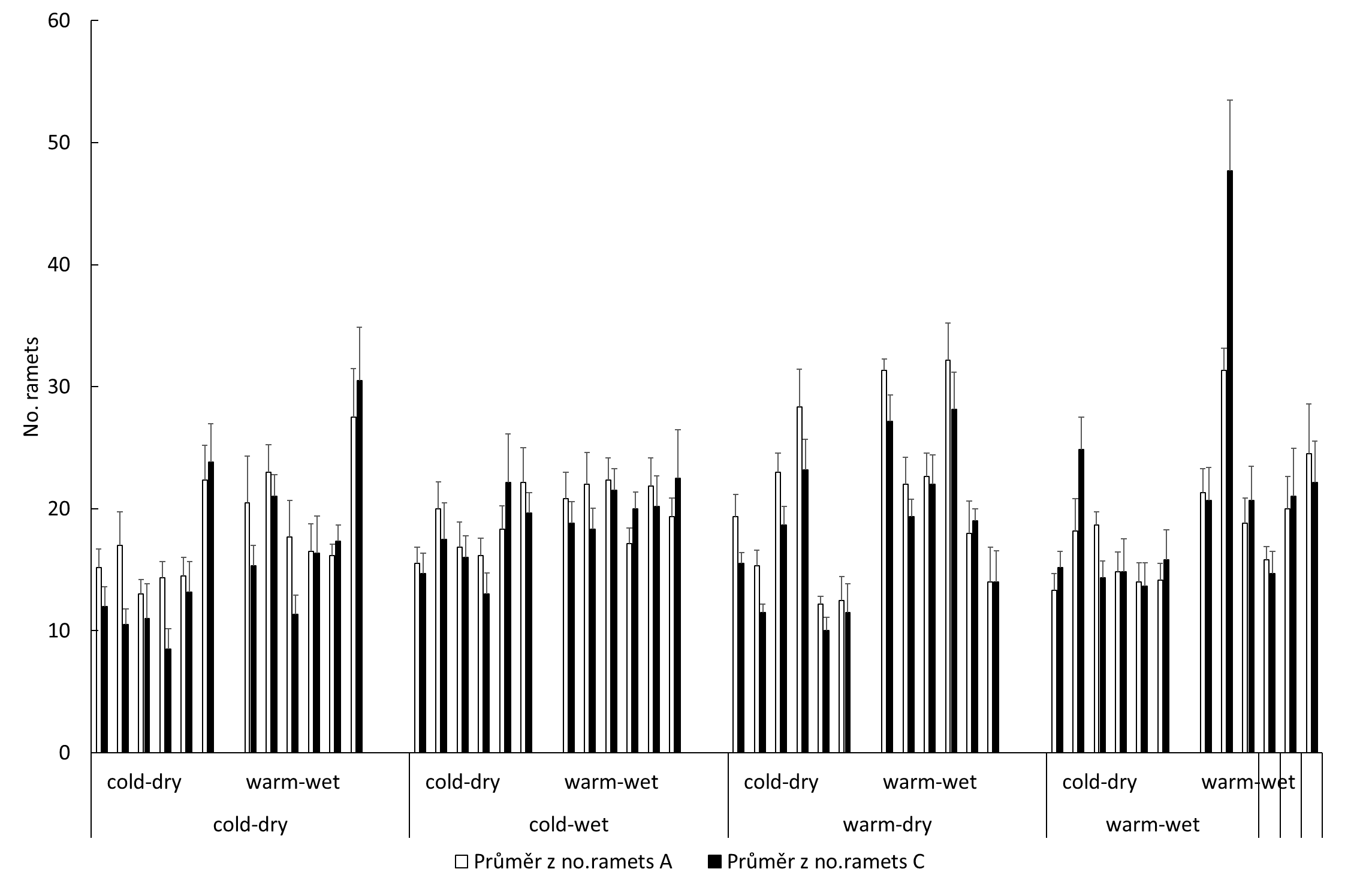


F)


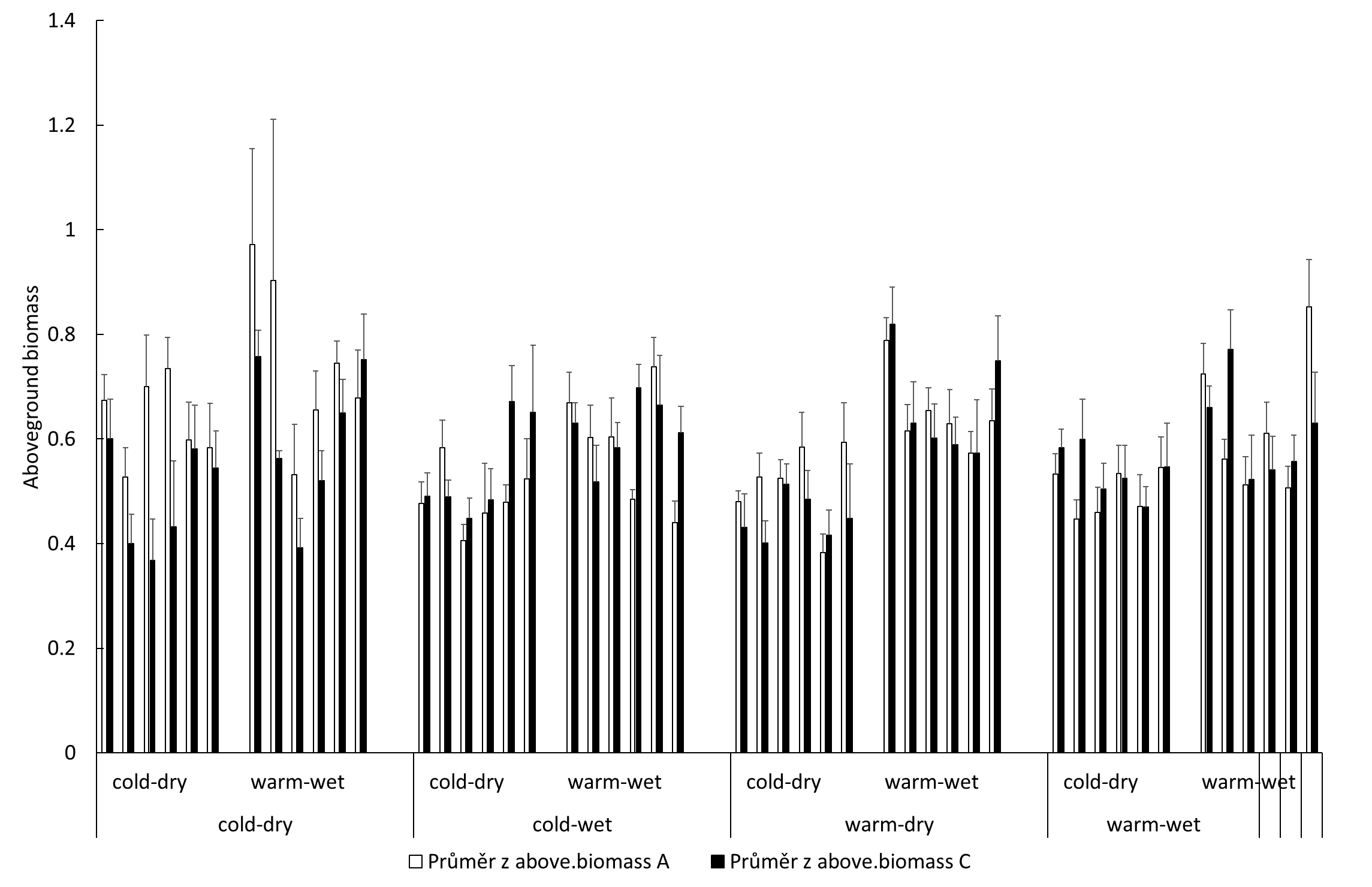

Supplement: Supplementary file 1 — Supplementary Information. [file 41598_2022_22125_MOESM1_ESM.docx]
